# The SMAD2/miR-4256/HDAC5/p16^INK4a^ signaling axis contributes to gastric cancer progression

**DOI:** 10.32604/or.2023.029101

**Published:** 2023-06-27

**Authors:** MIN WANG, HAILIANG ZHAO, WEIWEI CHEN, CAIQUN BIE, JINYING YANG, WENRUI CAI, CHUTIAN WU, YANFANG CHEN, SHUFEN FENG, YING SHI, YUTING LI, HUIJUN TANG, LIXIAN ZHONG, LILIANGZI GUO, SISI CHEN, LINJING LONG, SHAOHUI TANG

**Affiliations:** 1 Department of Gastroenterology, The First Affiliated Hospital, Jinan University, Guangzhou, 510632, China; 2 Department of Gastroenterology, Affiliated Hospital of Youjiang Medical University for Nationalities, Baise, 533000, China; 3 Department of Gastroenterology, The First People’s Hospital of Zunyi, The Third Affiliated Hospital of Zunyi Medical University, Zunyi, 563099, China; 4 Department of Gastroenterology, Shenzhen Hospital of Integrated Traditional Chinese and Western Medicine, Shenzhen, 518104, China; 5 Department of Gastroenterology, The Fifth Affiliated Hospital of Guangzhou Medical University, Guangzhou, 510799, China

**Keywords:** Gastric cancer, miR-4256, SMAD2, HDAC5, p16^INK4a^

## Abstract

The dysregulation of exosomal microRNAs (miRNAs) plays a crucial role in the development and progression of cancer. This study investigated the role of a newly identified serum exosomal miRNA miR-4256 in gastric cancer (GC) and the underlying mechanisms. The differentially expressed miRNAs were firstly identified in serum exosomes of GC patients and healthy individuals using next-generation sequencing and bioinformatics. Next, the expression of serum exosomal miR-4256 was analyzed in GC cells and GC tissues, and the role of miR-4256 in GC was investigated by *in vitro* and *in vivo* experiments. Then, the effect of miR-4256 on its downstream target genes HDAC5/p16^INK4a^ was studied in GC cells, and the underlying mechanisms were evaluated using dual luciferase reporter assay and Chromatin Immunoprecipitation (ChIP). Additionally, the role of the miR-4256/HDAC5/p16^INK4a^ axis in GC was studied using *in vitro* and *in vivo* experiments. Finally, the upstream regulators SMAD2/p300 that regulate miR-4256 expression and their role in GC were explored using *in vitro* experiments. miR-4256 was the most significantly upregulated miRNA and was overexpressed in GC cell lines and GC tissues; *in vitro* and *in vivo* results showed that miR-4256 promoted GC growth and progression. Mechanistically, miR-4256 enhanced HDAC5 expression by targeting the promoter of the HDAC5 gene in GC cells, and then restrained the expression of p16^INK4a^ through the epigenetic modulation of HDAC5 at the p16^INK4a^ promoter. Furthermore, miR-4256 overexpression was positively regulated by the SMAD2/p300 complex in GC cells. Our data indicate that miR-4256 functions as an oncogene in GC via the SMAD2/miR-4256/HDAC5/p16^INK4a^ axis, which participates in GC progression and provides novel therapeutic and prognostic biomarkers for GC.

## Introduction

Gastric cancer (GC) is the fourth most common malignancy by incidence, and the third most common malignancy by mortality worldwide [1]. Although many advancements have been achieved in terms of surgery, chemotherapy, radiotherapy and targeted therapy strategies, the overall prognosis for GC patients has remained largely unsatisfactory over the past few decades [2]. The 5-year overall survival from GC is less than 30% in most countries [3]. One of the major obstacles in improving the survival rate of GC patients is the lack of reliable noninvasive biomarkers for gastric cancer diagnosis, prognosis and therapy [4]. Therefore, there is an urgent need to gain detailed knowledge of GC initiation and progression, and develop novel effective prognostic biomarkers and therapeutic targets for GC.

Exosomes, membrane-bound vesicles 40–100 nm in diameter, are released from most cell types and are present in almost all biological fluids [5]. They are considered as a bridge for information exchange between cells by transporting proteins, lipids and nucleic acids to neighboring or distant cells [6]. Recently, microRNAs (miRNAs), a class of 18–24 nt small noncoding RNAs that generally posttranscriptionally regulate gene expression, have been identified in exosomes [7]. Moreover, cancer cells are reported to secrete at least 10-fold more exosomes than normal cells, and tumor-derived exosomes (TEXs) contain a unique expression profile of mRNAs and miRNAs that is different from normal cells [8,9]. Growing evidence suggests that exosomal miRNAs have a significant function in the onset and progression of GC, including tumor growth, angiogenesis, metastasis and chemoresistance [10–13]. For instance, exosomal miR-27a was found to induce the reprogramming of fibroblasts into cancer-associated fibroblasts in GC and promote GC cell proliferation, migration, and invasion [11]. Additionally, exosomal miR-423-5p has been found to enhance GC cell proliferation and migration, and contribute to poor outcomes in GC patients [12]. The latest evidence has shown that exosomal miR-107 can reverse chemotherapeutic drug resistance in GC cells [13]. Exosomal miRNAs have been proposed as an attractive tool for clinical tumor diagnosis, prognosis and therapy due to their features of quick detection, convenient collection, high stability and enrichment in circulating blood [14,15]. Therefore, the study of the functions and mechanism of exosomal miRNAs in the occurrence or metastasis of GC would help improve the clinical outcome of GC patients.

In this work, next generation sequencing was performed to identify differentially expressed miRNAs in serum exosomes from 7 GC patients and 2 healthy controls. We found that a total of 218 miRNAs were differentially expressed in GC. Next, potential target genes and biological functions that might be affected by these miRNAs were identified by performing a bioinformatics analysis. Importantly, a miRNA, hsa-miR-4256 (NCBI Entrez Gene: 100422976, hereafter designated miR-4256), which is 64 nucleotides in length, located on human chromosome 1p13.2 and was the most significantly upregulated miRNA among 218 miRNAs, attracted our attention as it has not been characterized in human diseases including GC. Therefore, in this study, we further explored the role of miR-4256 in GC tumorigenesis, and probed the mechanism of miR-4256 overexpression and its downstream molecular pathway to identify novel reliable and convenient biomarkers for GC prognosis and therapy. up-regulated.

## Materials and Methods

### Sample collection

Serum samples from 7 GC patients and 2 healthy controls (HC) who had no history of basic or chronic diseases, and 36 tumor tissues and matched adjacent nontumor tissues from GC patients who did not receive radiotherapy and chemotherapy before surgery were collected from the First Affiliated Hospital of Jinan University. Samples were stored at −80°C until use. Informed consent was obtained for all individuals. Ethics approval was obtained from the Ethics Committee of The First Affiliated Hospital of Jinan University.

### Exosomal RNA extraction and RNA library preparation

Exosomes were first isolated from serum using ExoQuick™ (System Biosciences, USA) according to the manufacturer’s instructions. Briefly, 300 μl of cell-free serum samples were transferred to a tube and mixed with 75 μl of ExoQuick reagent. Next, the mixture was incubated at 4°C overnight and then centrifuged at 1500 g for 30 min. Then, the pelleted exosomes were resuspended in 40 μl PBS. Finally, RNA of exosomes was extracted with the miRNeasy micro kit (Qiagen, USA) based on the manufacturer’s instructions.

### Sequencing data analysis

Next-generation sequencing was performed on an Illumina HiSeq2000 platform by Haplox, Inc. (Nanjing, China). The clustering of the index-coded samples was first carried out on a cBot Cluster Generation System using the TruSeq SR Cluster kit v3-cBot-HS (Illumina, USA). Then, the libraries were sequenced and 50 bp single-end reads were generated. Next, clean data were acquired by eliminating the low quality reads and adaptors in HTseq-count (version 0.7.2), FastQC (version 0.11.7) and Trimmomatic (version 0.38) software. Finally, clean reads were mapped to the reference sequence using Bowtie2 (version 2.3.4.1) from miRBase (Release 22) and NCBI human genome reference sequences, and used to analyze the miRNA profile from the clean data. The R package DESeq2 (version 1.20.0) was used to identify differentially expressed miRNAs. The starBase database (http://starbase.sysu.edu.cn/) was used to identify target genes of differentially expressed miRNAs. Gene Ontology (GO) function and Kyoto Encyclopedia of Genes and Genomes (KEGG) pathway enrichment analyses were applied to predict the biological function of mRNAs. GO functional analysis included three functional groups: biological processes (BP), molecular functions (MF), and cellular components (CC). The GO and KEGG analyses were performed in the R package clusterProfiler.

### Cell lines and cell culture

Human GC cell lines HGC-27, SGC-7901 and AGS, normal gastric epithelial cell line GES-1 were purchased from American Type Culture Collection (Manassas, USA). The cells were cultured in RPMI-1640 medium (Gibco, USA) supplemented with 10% fetal bovine serum (Gibco) in a humidified incubator at 37°C in 5% CO_2_.

### Quantitative real-time RT‒PCR

Total RNA was extracted from the cultured cells and tissues using TRIzol Reagent (Invitrogen, USA) following the manufacturer’s protocols. The mRNA and miRNA levels were quantified using Power SYBR Green PCR Master Mix (Applied Biosystems, USA) and TaqMan microRNA assay kits (Applied Biosystems), respectively. GAPDH was used as an internal control for mRNA, and U6 small nuclear RNA (snRNA) was used as an internal control for miRNA. Each experiment was repeated three times, and all reactions were carried out in triplicate.

### Cell transfection

Cells were seeded in 24-well plates, incubated for 24 h, and then transfected with miR-4256 mimic and miR-4256 inhibitor (Dharmacon, USA), small interfering RNAs (siRNAs) for HDAC5 (siHDAC5), HDAC5 expression plasmids, siRNAs for SMAD2 (siSMAD2) and SMAD2 expression plasmids (Invitrogen) using Lipofectamine 2000 (Invitrogen) based on the manufacturer’s instructions. Cells were collected 48 h after transfection for analysis. The HDAC5 and SMAD2 expression plasmids were synthesized and constructed by Synbio Technologies (Suzhou, China).

### Cell counting kit-8 (CCK-8) assay

The proliferation rate of cells was evaluated using the Cell Counting Kit-8 assay (Dojindo, Japan). Cells were seeded in 96-well plates for 24, 48 or 72 h, and 10 μL of CCK-8 reaction mixture was added into each well at the same time every day. After 4 h of incubation, the absorbance at a wavelength of 450 nm was evaluated by an automatic microplate reader (BioTek, USA).

### Transwell cell migration and invasion assays

The difference between the methods used for the migration assay and invasion assay was that the upper chamber for the invasion assay was covered with Matrigel (BD Biosciences, USA). Briefly, serum-free RPMI-1640 (Gibco) with 10% fetal bovine serum was placed in the bottom chamber. Then, cells were seeded into the upper chamber with serum-free RPMI 1640. After 24 h of incubation at 37°C, we used methanol to fix the cells within the membrane and stained them with crystal violet. Finally, the migrated or invaded cells were evaluated with a microscope.

### Establishment of tumor xenografts in nude mice

SGC-7901 cells (5 × 10^6^ cells/mouse) were subcutaneously injected into BALB/c nude mice to establish subcutaneous human GC xenograft models. After 7 days, the transplanted nude mice were randomly divided into three groups (Groups I–III). The miR-4256 agomir, miR-4256 antogomir, or scrambled control (RiboBio Co., Ltd., Guangzhou, China) was directly injected into the implanted tumor in Groups I–III at a dose of 2.5 nmol per mouse every 4 days for a total of five injections as previously described [16]. Moreover, we conducted another animal experiment, in which BALB/c nude mice were randomly divided into four groups (Groups I–IV). SGC-7901 cells (5 × 10^6^ cells/mouse) were subcutaneously injected into BALB/c nude mice in Groups I–III, and SGC-7901 cells stably transfected with pcDNA-p16^INK4a^ (5 × 10^6^ cells/mouse) were injected into nude mice in Group IV to establish subcutaneous GC xenograft models. After 7 days, scrambled control, miR-4256 agomir, miR-4256 agomir+cholesterol-modified siHDAC5, and miR-4256 agomir+cholesterol-modified siHDAC5 were intratumorally injected in Groups I–IV at a dose of 2.5 nmol per mouse for the scrambled control and miR-4256 agomir and at a dose of 0.1 nmol per mouse for cholesterol-modified siHDAC5 every 4 days for five total injections. Tumor size was monitored and measured with calipers every 4 days, and tumor volume was calculated by the formula (L × W × W/2), where L represents length, and W represents width. The animal experiments acquired ethical authorization from the Animal Research Ethics Committee of Jinan University.

### Luciferase reporter assay

The HDAC5 promoters (−1241/+11) of the human HDAC5 gene (hg38_chr17:44078376-44125275) containing the wild type (WT) or mutant type (MUT) miR-4256 binding sequences were synthesized by Synbio Technologies. Then, they were cloned into pGL4.10 luciferase reporter vector (Promega) to generate pGL4-HDAC5-WT and pGL4-HDAC5-MUT vectors. All the constructs were verified by sequencing. A total of 5 × 10^5^ cells were seeded in 24-well plates. Cells were cotransfected with 0.12 μg of either pGL4-HDAC5-WT reporter or pGL4-HDAC5-MUT reporter together with miR-4256 mimics or scrambled control (Dharmacon) and pRL-SV40 vector (Promega) for normalization of transfection efficiency using Lipofectamine 2000 (Invitrogen) following the manufacturer’s protocol. After 48 h of transfection, luciferase activity was measured utilizing a Luciferase Assay System (Promega).

The miR-4256 promoters (−1000/−1) of the human miR-4256 gene (hg38_chr1:112461834-112462833) containing the wild-type or three mutant types (MUT1+2 (site 1+2), MUT1(site 1), and MUT2 (site 2)) of SMAD2 binding sequences were synthesized by Synbio Technologies. Then, they were cloned into the pGL4.10 luciferase reporter vector (Promega, USA) to generate the pGL4-miR-4256-WT reporter, pGL4-miR-4256-MUT1+2 reporter, pGL4-miR-4256-MUT1 reporter, and pGL4-miR-4256-MUT2 reporter. All the constructs were verified by sequencing. A total of 5 × 10^5^ cells were seeded in 24-well plates. Cells were cotransfected with 0.12 μg of pGL4-miR-4256-WT reporter, pGL4-miR-4256-MUT1+2 reporter, pGL4-miR-4256-MUT1 reporter, or pGL4-miR-4256-MUT2 reporter together with the SMAD2 expression plasmid or SMAD2 negative control and pRL-SV40 vector (Promega) for normalization of transfection efficiency using Lipofectamine 2000 (Invitrogen) following the manufacturer’s protocol. After 48 h of transfection, luciferase activity was measured utilizing a Luciferase Assay System (Promega).

### Western blot

The determination of protein concentration was performed with a BCA Assay Kit (Thermo Fisher Scientific, USA) as instructed by the manufacturer. Next, 10% sodium dodecyl sulfate‒polyacrylamide gel electrophoresis (Millipore, USA) was used to separate the protein samples. Then, the samples were transferred onto polyvinylidene difluoride (Millipore) membranes. Thereafter, the membranes were blocked with 5% nonfat milk at room temperature for 2 h and incubated at 4°C overnight with primary antibodies, including anti-HDAC5 (Abcam, UK), anti-p16^INK4a^ (Abcam), anti-SMAD2 (Abcam) and anti-p300 (Abcam) and anti-GAPDH (Abcam) antibodies. After incubation with secondary antibodies and washing, the target bands were visualized using an enhanced chemiluminescence detection system (Amersham Biosciences, USA). The samples were normalized to the internal control GAPDH.

### Chromatin immunoprecipitation (ChIP) assays

The ChIP assays were performed as standard operation procedure. Briefly, formaldehyde was first used to fix the cells. Next, the chromatin was sonicated into fragments at 200–1000 bp. Then, the chromatin was incubated and precipitated with antibodies against H3K27ac (Millipore), H4K16ac (Millipore), HDAC5 (Abcam), SMAD2 (Abcam), p300 (Abcam) or IgG (Abcam). The amount of immunoprecipitated DNA was calculated in reference to a standard curve and normalized to input DNA.

### Coimmunoprecipitation (Co-IP) assays

Coimmunoprecipitation assays were performed by protein A/G conjugated magnetic beads (Invitrogen). The lysates of cells were immunoprecipitated with anti-SMAD2 (Millipore) or anti-p300 (Millipore) antibodies overnight at 4°C. Protein bindings were detected by western blotting with the respective antibodies.

### Statistical analysis

We performed our experiments in triplicate, and the results are presented as the mean value ± standard deviation. We statistically analyzed the categorical data with χ2 or Fisher’s exact tests and quantitative data with Student’s *t* test using SPSS statistical software. Linear correlation was evaluated by Pearson correlation coefficient, and *p* < 0.05 was considered statistically significant.

## Results

### miRNA expression profiles in serum exosomes of GC patients

**Figure S1:**
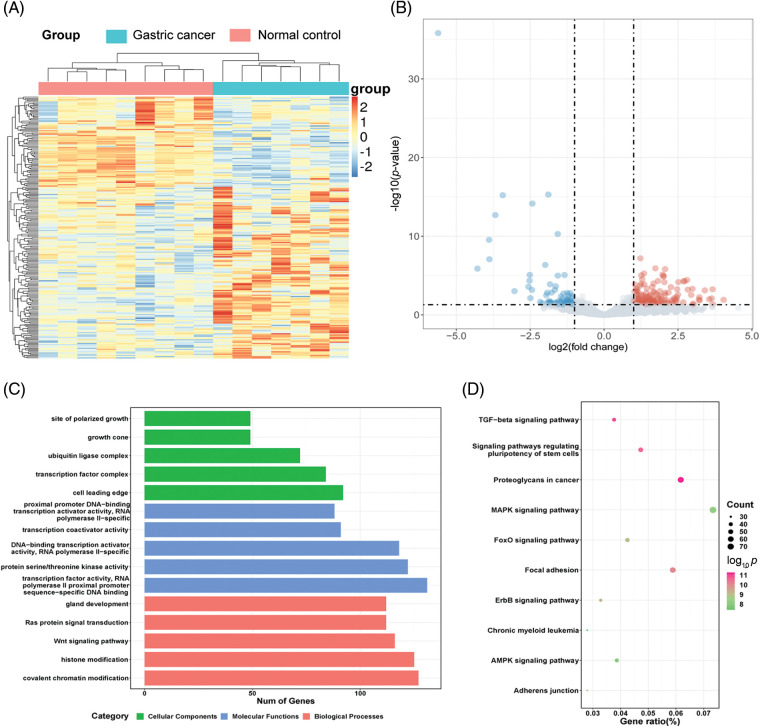
The miRNA expression profiles in the serum exosomes of GC patients. (A) Heat map showed the miRNA expression profile. (B) Volcano plot showed differentially expressed miRNAs in serum exosomes of GC patients and normal controls. (C) The top 5 GO items of the predicted targets of the upregulated miRNAs were displayed in the bar plot. (D) The top 10 KEGG pathways of the target genes of the upregulated miRNAs were displayed in the bubble chart.

**Figure S2:**
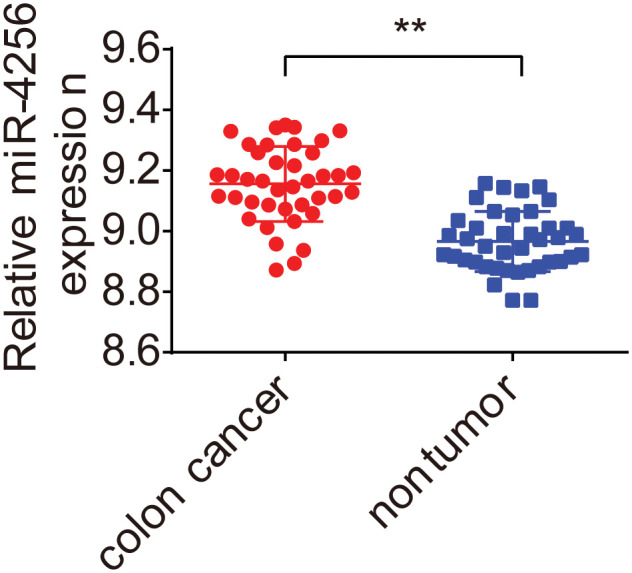
miR-4256 expression was increased in colon cancer tissues. The expression levels of miR-4256 in 40 human colon cancer tissues and 40 matched adjacent nontumor colon tissues (GSE49246) were downloaded from the public functional genomics data repository-Gene Expression Omnibus database (GEO, http://www.ncbi.nlm.nih.gov/geo) of the National Center for Biotechnology Information (NCBI). **p < 0.01.

To evaluate the miRNA expression profiles in serum exosomes of GC patients, we performed next-generation sequencing using serum exosomes of 7 GC patients and 2 healthy individuals. We detected differentially expressed miRNAs using the following criteria with log2 (fold change) ≥ 1 and *p* ≤ 0.05 (Suppl. Fig. S1A). The primary data are shown in a volcano plot (Suppl. Fig. S1B). We found that 218 miRNAs were differentially expressed in GC samples, of which 139 miRNAs were upregulated and 79 miRNAs were downregulated. The top 10 significantly upregulated miRNAs in the GC samples are listed in Table 1. Among the differentially expressed miRNAs, we focused on miR-4256 because it was the most differentially expressed miRNA among the 139 miRNAs upregulated in the serum exosomes of GC patients. We searched the PubMed database and found that the roles of miR-4256 in human diseases, including cancer, have not been studied. By further searching the Gene Expression Omnibus (GEO) database (http://www.ncbi.nlm.nih.gov/geo) and The Cancer Genome Atlas (TCGA) database (http://cancergenome.nih.gov/), we only found that the expression level of miR-4256 was higher in 40 colon cancer tissues than in 40 paired adjacent nontumor tissues (Suppl. Fig. S2), indicating that miR-4256 may exert oncogenic activities. Thus, miR-4256 was selected for further investigation.

**Table 1:** The top 10 significantly upregulated miRNAs in GC patients

miRNA	log2 (fold change)	*p* value
hsa-miR-4256	3.696959	0.000189
hsa-miR-548m	3.415406	0.000362
hsa-miR-520e-3p	3.260379	0.002319
hsa-miR-891a-3p	2.991121	0.000364
hsa-miR-6745	2.941745	0.002014
hsa-miR-539-5p	2.846582	0.000292
hsa-miR-3119	2.805013	1.70E-05
hsa-miR-8076	2.710387	4.89E-05
hsa-miR-6727-5p	2.688992	5.18E-05
hsa-miR-6733-3p	2.518561	4.78E-05

### Target gene prediction and GO and KEGG pathway enrichment of the predicted genes

The starBase database (http://starbase.sysu.edu.cn/) was employed to predict target genes of 218 differentially expressed miRNAs. As a result, 3401 genes were identified as predicted targets of the upregulated miRNAs, and 16823 genes were predicted as the target genes of the downregulated miRNAs. To comprehensively describe the properties of the putative target genes, Gene Ontology (GO) and Kyoto Encyclopedia of Genes and Genomes (KEGG) pathway enrichments were performed by the R package clusterProfiler. GO analysis was divided into three functional groups, including molecular function, biological processes, and cell composition. For the biological processes, histone modification and covalent chromatin modification were significantly enriched; for the cell composition, cell leading edge was dominant, and for the molecular function, transcription factor activity/RNA polymerase II proximal promoter sequence-specific DNA binding was more abundant (Suppl. Fig. S1C). KEGG analysis results indicated that the MAPK signaling pathway and proteoglycans in the cancer pathway were more abundant (Suppl. Fig. S1D).

### miR-4256 is overexpressed in GC cell lines and GC tissues

**Figure 1:**
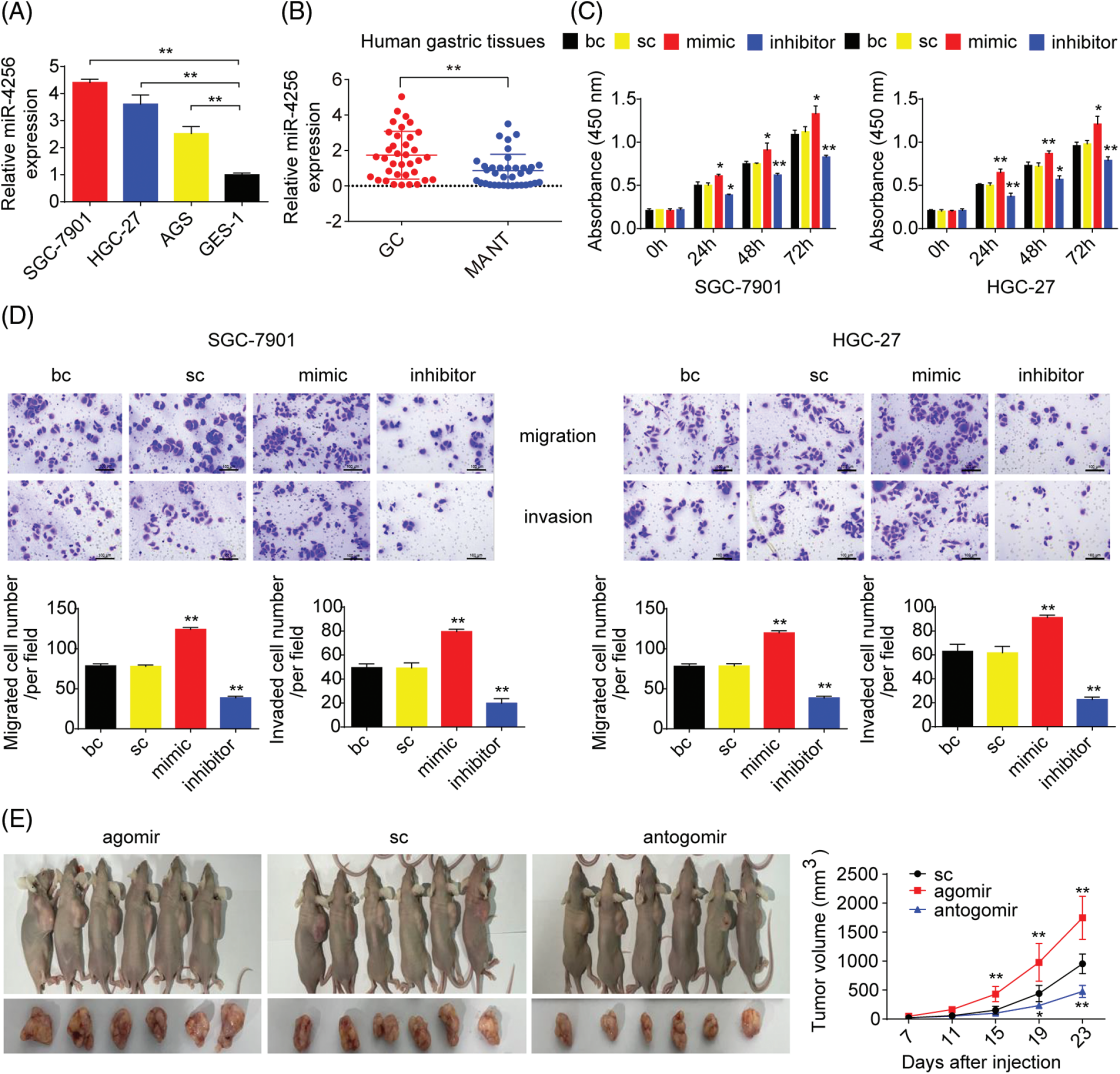
miR-4256 was overexpressed in GC and promoted GC growth and invasion *in vitro* and *in vivo*. (A) The expression levels of miR-4256 were detected in GC cells (SGC-7901, HGC-27, and AGS) and the human gastric epithelial cell line (GES-1) by RT‒qPCR. **p < 0.01 *vs.* GES-1 cells. (B) The expression levels of miR-4256 were detected in 36 human GC tissues and 36 MANT by RT‒qPCR. **p < 0.01 *vs.* MANT. (C) Effects of miR-4256 mimics or inhibitors on the proliferation of SGC-7901 and HGC-27 cells were determined by CCK-8 assays. **p < 0.01, *p < 0.05 *vs.* bc or sc. (D) Effects of miR-4256 mimics or inhibitors on the migration and invasion of SGC-7901 and HGC-27 cells were determined by Transwell assays. **p < 0.01 *vs.* bc or sc. (E) Tumour size was monitored and measured with calipers starting from the 7th day after tumour cell inoculation in each group. The pictures of xenograft tumors and grow curves of tumors from miR-4256 agomir, miR-4256 antogomir and sc group. **p < 0.01 *vs.* sc. GC, gastric cancer tissues; MANT, matched adjacent nontumor tissues; bc, blank control; sc, scrambled control; mimic, miR-4256 mimic; inhibitor, miR-4256 inhibitor; agomir, miR-4256 agomir; antogomir, miR-4256 antogomir. Each experiment was repeated three times.

To measure the expression of miR-4256, we performed RT‒qPCR and found that miR-4256 expression was significantly upregulated in the GC cell lines SGC-7901, HGC-27, and AGS compared with that in the human gastric epithelial cell line GES-1 (Fig. 1A). Then, human stomach tissue samples were examined, and RT‒qPCR analysis revealed that miR-4256 expression was also significantly upregulated in human GC tissues compared with their matched adjacent nontumor tissues (MANT) (Fig. 1B). In addition, the correlation of the miR-4256 expression level with clinicopathological characteristics was investigated in GC patients. In 36 GC samples, the miR-4256 level for the cases with lymph node metastasis, later TNM stages (stage III-IV), and poorly differentiated GC tissues was significantly higher than that for cases without lymph node metastasis, with earlier TNM stages (stages I–II), and with moderately/well-differentiated GC tissues. However, no correlation was found between the miR-4256 level and the remaining clinicopathological parameters including peritoneal metastasis and venous invasion (*p* > 0.05) (Table 2). These results suggest the possibility that miR-4256 is involved in the development and progression of GC.

**Table 2:** Correlation of relative miR-4256 expression levels with clinicopathological features in 36 patients with gastric cancer

Parameters	No. of patients	Relative miR-4256 expression
Lymph node metastasis		
Negative	14	0.57 (0.23–1.93)*
Positive	22	1.90 (1.12–3.03)
TNM stage		
I–II stage	17	1.21 (0.19–1.79)*
III–IV stage	19	2.05 (0.83–3.32)
Peritoneal metastasis		
Negative	25	1.43 (0.41–2.04)
Positive	11	2.32 (0.61–3.59)
Venous invasion		
Negative	23	1.64 (0.36–2.83)
Positive	13	1.25 (0.56–2.68)
Tumor differentiation degree		
Moderately/well differentiated	12	0.42 (0.08–1.52)*
Poorly differentiated	24	1.93 (0.93–3.04)

Note: Data are shown as median (IQR) and compared by Mann-Whitney U test. *p < 0.05 *vs* corresponding groups of the same parameters. IQR, interquartile range.

### miR-4256 promotes GC cell proliferation, migration, and invasion in vitro

To assess the functional effects of miR-4256 *in vitro*, we investigated whether miR-4256 regulated GC cell proliferation or migration and invasion using CCK-8 or Transwell assays, respectively. *In vitro* assays were performed by transfecting miR-4256 mimics, miR-4256 inhibitors, and scrambled control into GC cells. The CCK-8 assay indicated that the proliferation of SGC-7901 and HGC-27 cells could be significantly enhanced by miR-4256 mimics and suppressed by miR-4256 inhibitors compared with the blank control or the scrambled control (Fig. 1C). Furthermore, Transwell assays revealed that the ectopic overexpression of miR-4256 promoted the migration and invasion of SGC-7901 and HGC-27 cells, and the suppression of endogenous miR-4256 expression reduced the migration and invasion of SGC-7901 and HGC-27 cells compared with the blank control or the scrambled control (Fig. 1D). Taken together, these observations indicate that miR-4256 can promote GC cell proliferation, migration and invasion in tumorigenesis.

### miR-4256 facilitates GC cell-derived xenograft tumor growth in nude mice

To determine the *in vivo* relevance of the aforementioned *in vitro* findings, the effect of miR-4256 on GC cell-derived xenograft tumorigenicity was next investigated. The tumors in the mice with miR-4256 agomir treatment or miR-4256 antogomir treatment grew more rapidly or less rapidly, respectively, compared with the scrambled control (Fig. 1E). This finding suggests that miR-4256 promotes GC cell-derived xenograft tumor growth in nude mice.

### miR-4256 promotes HDAC5 expression to exacerbate malignant biological behavior by targeting the promoter of the HDAC5 gene in GC cells

**Figure 2:**
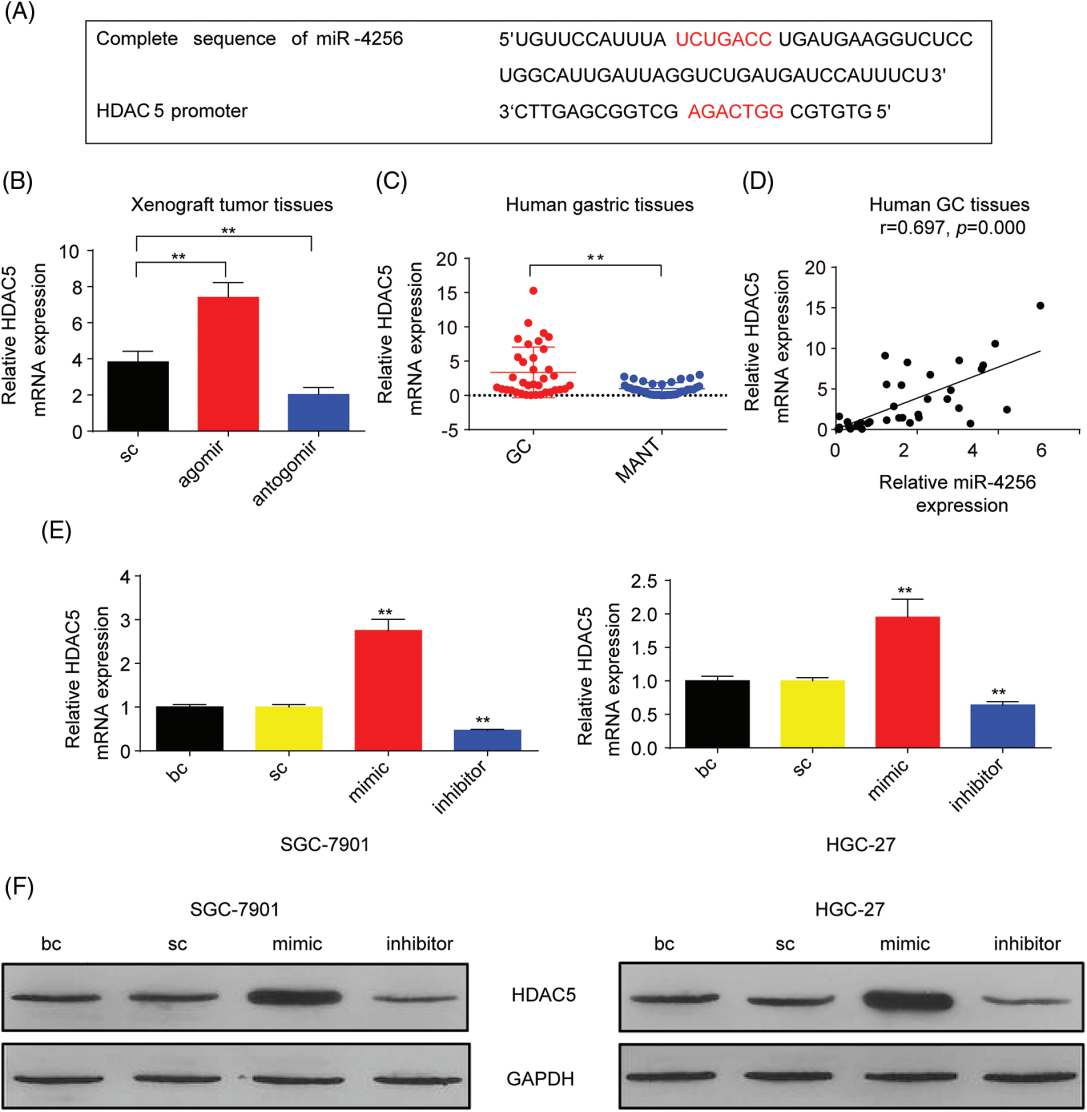
miR-4256 promoted HDAC5 expression in GC cells. (A) The complete sequence and the seed region sequence of human miR-4256 as well as its complementary sequence of the HDAC5 promoter. The seed region sequence and complementary sequence were marked in red. (B) The expression of HDAC5 was detected in xenograft tumor tissues from agomir, antogomir and sc group by RT‒qPCR. **p < 0.01 *vs.* sc. (C) The expression of HDAC5 was detected in 36 human GC tissues and 36 MANT by RT‒qPCR. **p < 0.01 *vs.* MANT. (D) HDAC5 expression was positively correlated with the miR-4256 expression in human GC tissues (r = 0.697, *p* = 0.000). (E, F) The expression of HDAC5 at both the mRNA and protein levels were detected by RT‒qPCR and western blot in SGC-7901 and HGC-27 cells transfected with mimics, inhibitors and sc. **p < 0.01 *vs.* bc or sc. agomir, miR-4256 agomir; antogomir, miR-4256 antogomir; GC, gastric cancer tissues; MANT, matched adjacent nontumor tissues; bc, blank control; sc, scrambled control; mimic, miR-4256 mimic; inhibitor, miR-4256 inhibitor. Each experiment was repeated three times.

**Figure S3:**
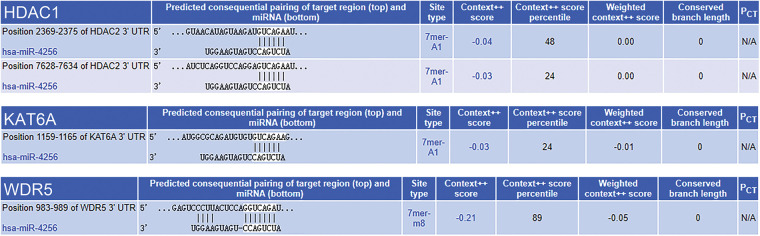
The predicted putative genes of miR-4256. The predicted miR-4256-binding sites in the 3′-UTR of three genes (WDR5, HDAC2 and KAT6A) by the Software miRTarBase, miRDB and TargetScan. UTR, untranslated regions.

Subsequently, the mechanism behind the oncogenic role of miR-4256 in 0GC was investigated. According to bioinformatics analysis, the target genes of miR-4256 may be involved in enzymes related to histone modification (ERHM), including histone acetyltransferases, histone deacetylases (HDACs), histone methyltransferases and histone demethylases. The miRTarBase, miRDB, and TargetScan databases were applied to predict the interaction between miR-4256 and the 3′-untranslated regions (3′-UTR) and 5′-UTR of mRNAs of the genes encoding human ERHM, and the results showed that there was a potential interaction between miR-4256 and the 3′-UTR of the mRNAs of three genes (WDR5, HDAC2 and KAT6A), suggesting that miR-4256 may downregulate their expression (Suppl. Fig. S3) and that their expression may be decreased in GC. However, these three genes have previously been reported to be overexpressed in cancer [17] or in GC [18,19] and exert oncogenic activities, which is inconsistent with our predicted results. Then, the potential binding sites of the gene promoter were further screened. DNA fragments (2 kb) upstream of the transcription start sites of the human ERHM genes were obtained from the NCBI database, and we found that there was a potential binding site for miR-4256 in the promoter of the HDAC5 gene (Fig. 2A).

To characterize the correlation between the expression of a potential target gene, HDAC5, and miR-4256, we examined HDAC5 mRNA in tumor tissues in nude mice by RT‒qPCR. As shown in Fig. 2B, the tumors in the mice with miR-4256 agomir treatment exhibited higher HDAC5 mRNA levels, whereas the tumors in the mice with miR-4256 antogomir treatment showed lower HDAC5 mRNA compared with the scrambled control. Subsequently, human gastric tissue specimens were examined and RT‒qPCR analysis revealed that HDAC5 mRNA expression was significantly upregulated in human GC tissues compared with that in their matched adjacent non-tumor tissues (Fig. 2C), which was in accordance with the aforementioned increased miR-4256 expression in human gastric tissues (Fig. 1B). Further correlation analysis demonstrated that the expression level of HDAC5 mRNA was positively correlated with miR-4256 expression (r = 0.697, *p* < 0.01) in human GC tissues (Fig. 2D). Furthermore, we transfected miR-4256 mimic, miR-4256 inhibitor, and scrambled control into SGC-7901 and HGC-27 cells. We found that the ectopic overexpression of miR-4256 increased the expression of HDAC5 mRNA, whereas the inhibition of endogenous miR-4256 expression resulted in downregulation of HDAC5 mRNA levels compared with the blank control or the scrambled control in the two cell lines (Fig. 2E). Moreover, these mRNA variations were translated into similar changes at the protein level (Fig. 2F).

**Figure 3:**
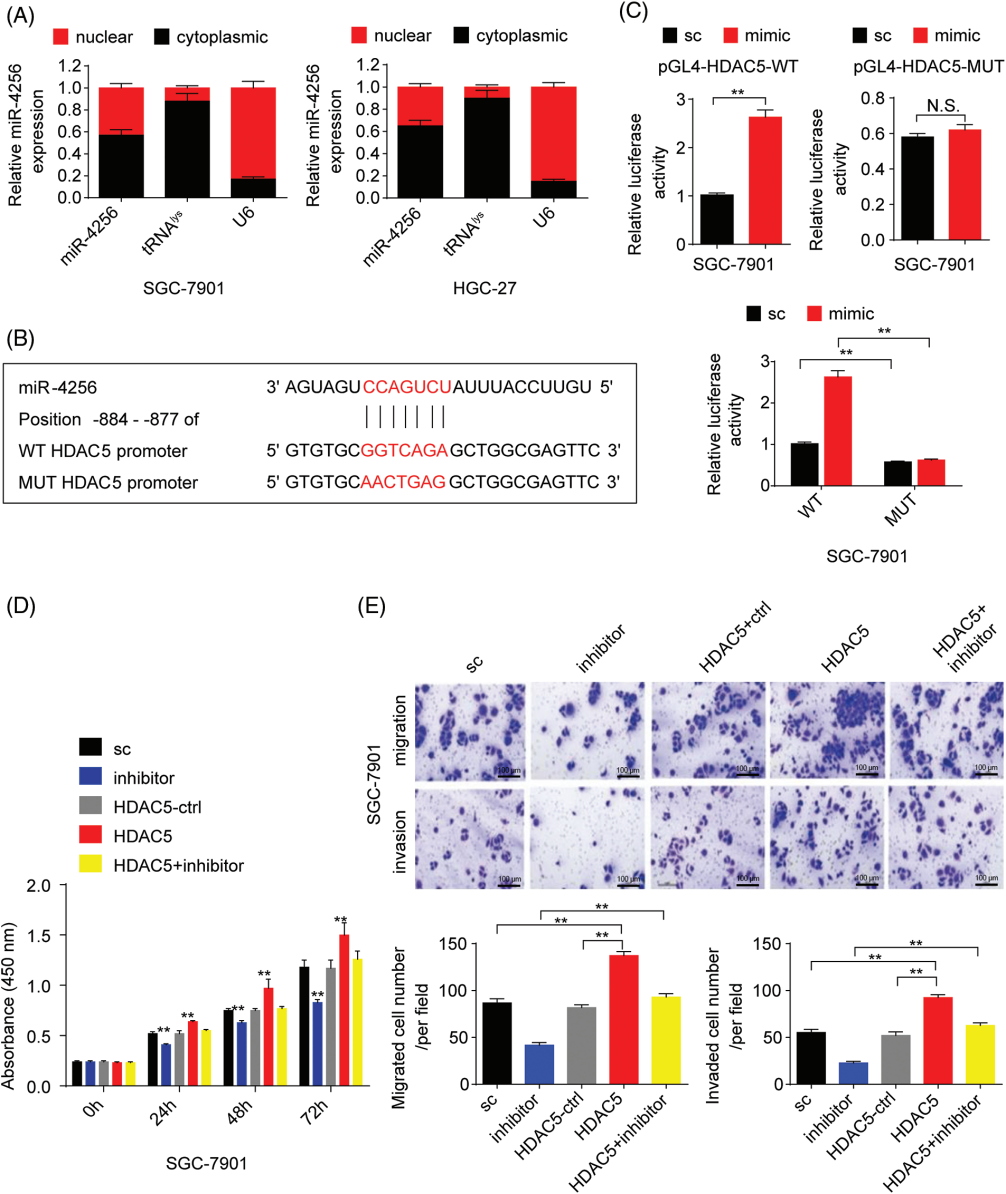
miR-4256 promoted GC cell proliferation and migration by targeting the promoter of the HDAC5 gene. (A) Subcellular fractionation location of miR-4256. The miR-4256 expression levels in the cell cytoplasm and nucleus of SGC-7901 and HGC-27 cells were detected by RT‒qPCR. Lysine-tRNA (tRNAlys) was used as a cytosol marker and U6 snRNA was used as a nucleus marker. (B) The partial sequence of human miR-4256 and the human HDAC5 promoter fragment containing wild-type (WT) or mutant (MUT) miR-4256-binding sequences. (C) Relative luciferase activities were evaluated in SGC-7901 cells cotransfected with the mimic or sc, and the pGL4-HDAC5-WT or pGL4-HDAC5-MUT. (D) Effects of miR-4256 inhibitor, HDAC5 and miR-4256 inhibitor plus HDAC5 on the proliferation of SGC-7901 cells were determined by CCK-8 assays. **p < 0.01 *vs.* sc, inhibitor or HDAC5-ctrl. (E) Effects of miR-4256 inhibitor, HDAC5 and miR-4256 inhibitor plus HDAC5 on the migration and invasion of SGC-7901 cells were determined by Transwell assays. **p < 0.01 *vs.* sc, inhibitor or HDAC5-ctrl. bc, blank control; sc, scrambled control; mimic, miR-4256 mimic; inhibitor, miR-4256 inhibitor; HDAC5, HDAC5 expression plasmid; HDAC5-ctrl, HDAC5-negative control; WT, pGL4-HDAC5-WT; MUT, pGL4-HDAC5-MUT. Each experiment was repeated three times.

**Figure S4:**
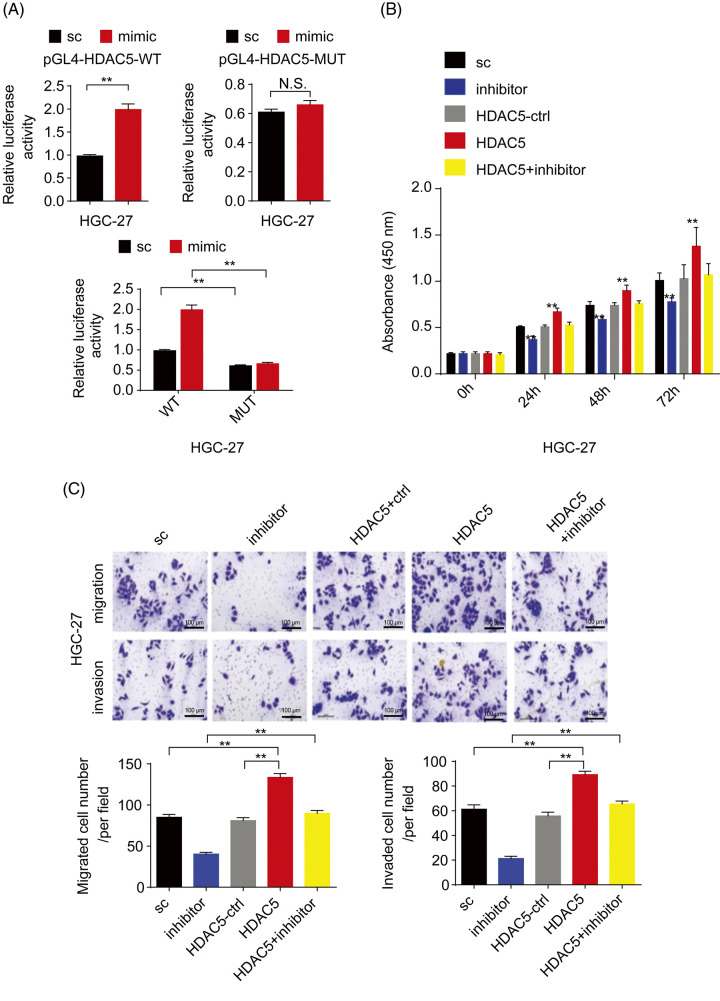
miR-4256 stimulated malignant biological behavior of GC cells by targeting the promoter of the HDAC5 gene. (A) Relative luciferase activities were evaluated in HGC-27 cells cotransfected with the mimic or sc, and the pGL4-HDAC5-WT or pGL4-HDAC5-MUT. (B) Effects of miR-4256 inhibitor, HDAC5 and miR-4256 inhibitor plus HDAC5 on the proliferation of HGC-27 cells were determined by CCK-8 assays. **p < 0.01 *vs.* sc, inhibitor or HDAC5-ctrl. (C) Effects of miR-4256 inhibitor, HDAC5 and miR-4256 inhibitor plus HDAC5 on the migration and invasion of HGC-27 cells were determined by Transwell assays. **p < 0.01 *vs.* sc, inhibitor or HDAC5-ctrl. bc, blank control; sc, scrambled control; mimic, miR-4256 mimic; inhibitor, miR-4256 inhibitor; HDAC5, HDAC5 expression plasmid; HDAC5-ctrl, HDAC5-negative control. Each experiment was repeated three times.

Furthermore, to confirm whether miR-4256 directly recognizes the promoter of the HDAC5 gene, the following assays were performed. First, miR-4256 expression was examined in the nuclear and cytoplasmic fractions of SGC-7901 and HGC-27 cells, and the results indicated that miR-4256 expression was detected in both the nucleus and cytoplasm (Fig. 3A), demonstrating that mature miR-4256 trafficked to the nucleus in the two cell lines. This is consistent with previous reports that showed that a fraction of miRNAs was located in the nucleus [20,21]. Then, a luciferase reporter assay was performed (Fig. 3B). The results indicated that compared with the scrambled control, the miR-4256 mimic induced a significant increase in the relative luciferase activity of the pGL4-HDAC5-WT reporter, but the relative luciferase activity of the pGL4-HDAC5-MUT reporter was not significantly affected by miR-4256 mimic transfection in GC cells (SGC-7901 and HGC-27 cells). In addition, the relative luciferase activity of the pGL4-HDAC5-WT reporter was significantly higher than that of the pGL4-HDAC5-MUT reporter in GC cells (SGC-7901 and HGC-27 cells) transfected with the scrambled control or miR-4256 mimic (Fig. 3C and Suppl. Fig. S4A).

Finally, we determined whether the effects of miR-4256 on GC cell proliferation, migration, and invasion are indeed mediated by HDAC5. CCK-8 and Transwell assays revealed that transient overexpression of HDAC5 exacerbated proliferation, migration, and invasion in GC cells compared with the HDAC5-negative control or the scrambled control; of note, the combination of transient transfection of HDAC5 and miR-4256 inhibitor eliminated the inhibition of malignant biological behavior caused by the miR-4256 inhibitor (Figs. 3D, 3E and Suppl. Figs. S4B, S4C).

Together, these data indicate that miR-4256 upregulates HDAC5 expression to aggravate malignant biological behavior by targeting the promoter of the HDAC5 gene in GC cells.

### miR-4256 restrains the expression of p16^*INK4a*^ to boost the malignant biological behavior of GC cells by the epigenetic modulation of HDAC5 at the p16^*INK4a*^ promoter

**Figure 4:**
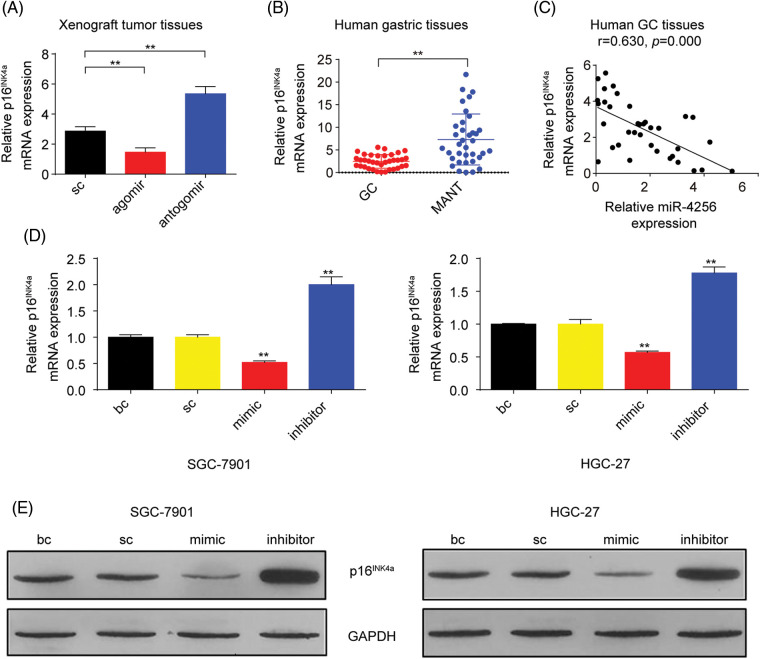
miR-4256 inhibited p16^INK4a^ expression in GC cells. (A) The expression of p16^INK4a^ was detected in xenograft tumor tissues from miR-4256 agomir, miR-4256 antogomir and scrambled control group by RT‒qPCR. **p < 0.01 *vs.* sc. (B) The expression levels of p16^INK4a^ were detected in 36 human GC tissues and 36 MANT by RT‒qPCR. **p < 0.01 *vs.* MANT. (C) p16^INK4a^ expression was negatively correlated with the miR-4256 expression in human GC tissues (r = 0.630, *p* = 0.000). (D, E) The expression of p16^INK4a^ at both the mRNA and protein levels were detected by RT‒qPCR and western blot in miR-4256 mimic, miR-4256 inhibitor, sc and bc. **p < 0.01 *vs.* sc or bc. agomir, miR-4256 agomir; antogomir, miR-4256 antogomir; GC, gastric cancer tissues; MANT, matched adjacent nontumor tissues; bc, blank control; sc, scrambled control; mimic, miR-4256 mimic; inhibitor, miR-4256 inhibitor. *p < 0.05, **p < 0.01. Each experiment was repeated three times.

p16^INK4a^, which is a cyclin-dependent kinase inhibitor, has been reported to constrain tumor growth and inhibit oncogenic phenotypes [22]. Additionally, our aforementioned data found that miR-4256 enhanced the proliferation and invasion of GC cells. Hence, we speculated that miR-4256 might exert oncogenic activities by regulating the expression of p16^INK4a^. We first analyzed the expression of p16^INK4a^ mRNA in the tumor tissues in the nude mice by RT‒qPCR. The results showed that the tumors in the mice with miR-4256 agomir treatment displayed lower p16^INK4a^ mRNA, whereas the tumors in the mice with miR-4256 antogomir treatment revealed the opposite expression changes in p16^INK4a^ mRNA compared with the scrambled control (Fig. 4A).

Next, RT‒qPCR results from the human gastric tissues indicated that p16^INK4a^ mRNA expression was significantly downregulated in human GC tissues compared with that in their matched adjacent nontumor tissues, and the correlation analysis found that the p16^INK4a^ mRNA level was negatively correlated with miR-4256 expression (r = −0.630, *p* < 0.01) (Figs. 4B and 4C).

Then, miR-4256 mimics, miR-4256 inhibitors, and scrambled control were transfected into SGC-7901 and HGC-27 cells, which indicated that the ectopic overexpression of miR-4256 reduced the expression of p16^INK4a^ mRNA, whereas the inhibition of endogenous miR-4256 expression led to upregulation of p16^INK4a^ mRNA levels compared with the blank control or the scrambled control in the two cell lines (Fig. 4D). The negative regulation of p16^INK4a^ protein expression by miR-4256 was further confirmed by Western blot analysis in the two cell lines (Fig. 4E).

To further explore the potential mechanism by which miR-4256 regulates p16^INK4a^ expression, three databases (TargetScan, miRTarBase, and miRDB) were utilized to predict whether miR-4256 can interact with p16^INK4a^. The results showed that miR-4256 did not bind to the 3′UTR or 5′UTR of p16^INK4a^ mRNA or the promoter of the p16^INK4a^ gene, suggesting that mir-4256 may not directly regulate p16^INK4a^ expression and that the regulatory effect observed in the present study may be indirect.

**Figure S5:**
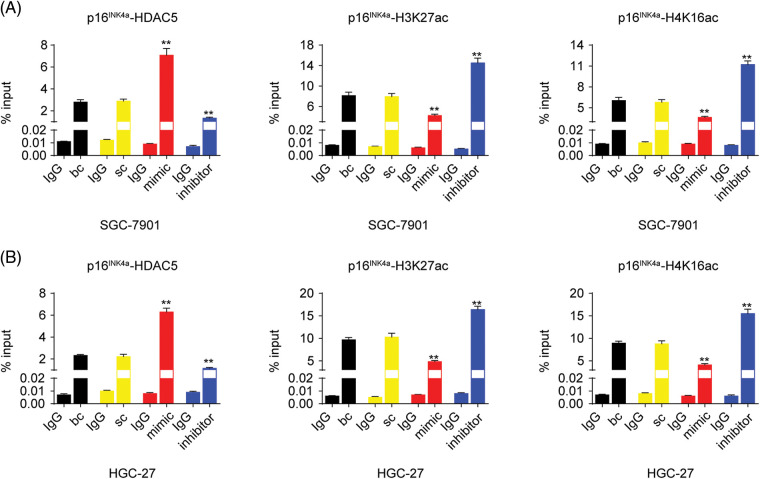
Effects of miR-4256 on the enrichment of HDAC5, H3K27ac, and H4K16ac at the promoter of the p16^INK4a^ gene in GC cells. (A) ChIP results of bindings of HDAC5, H3K27ac and H4Kac to the promoter of the p16^INK4a^ gene following miR-4256 mimic or miR-4256 inhibitor treatment in SGC-7901 cells. **p < 0.01 *vs.* bc or sc. (B) ChIP results of bindings of HDAC5, H3K27ac and H4Kac to the promoter of the p16^INK4a^ gene following miR-4256 mimic or miR-4256 inhibitor treatment in HGC-27 cells. **p < 0.01 *vs.* bc or sc. bc, blank control; sc, scrambled control; mimic, miR-4256 mimic; inhibitor, miR-4256 inhibitor. Each experiment was repeated three times.

As described above, our results indicated that miR-4256 both upregulated HDAC5 expression and downregulated p16^INK4a^ expression; additionally, HDACs are capable of deacetylating the lysine residues of histones and inhibiting gene expression, and aberrant expression of HDACs is associated with the development of various cancers [23]. Therefore, we determined whether miR-4256 regulated p16^INK4a^ expression via the epigenetic regulation mechanism involved in HDAC5. ChIP assays showed that the increased enrichment of HDAC5 and the decreased enrichment of activating histone marks histone 3 lysine 27 acetylation (H3K27ac) and histone 4 lysine 16 acetylation (H4K16ac) were detected at the p16^INK4a^ promoter following treatment with miR-4256 mimic, whereas treatment with miR-4256 inhibitor decreased the enrichment of HDAC5 and increased enrichment of the activating histone marks H3K27ac and H4K16ac compared with the blank control or the scrambled control in SGC-7901 cells (Suppl. Fig. S5A) and HGC-27 cells (Suppl. Fig. S5B).

**Figure 5:**
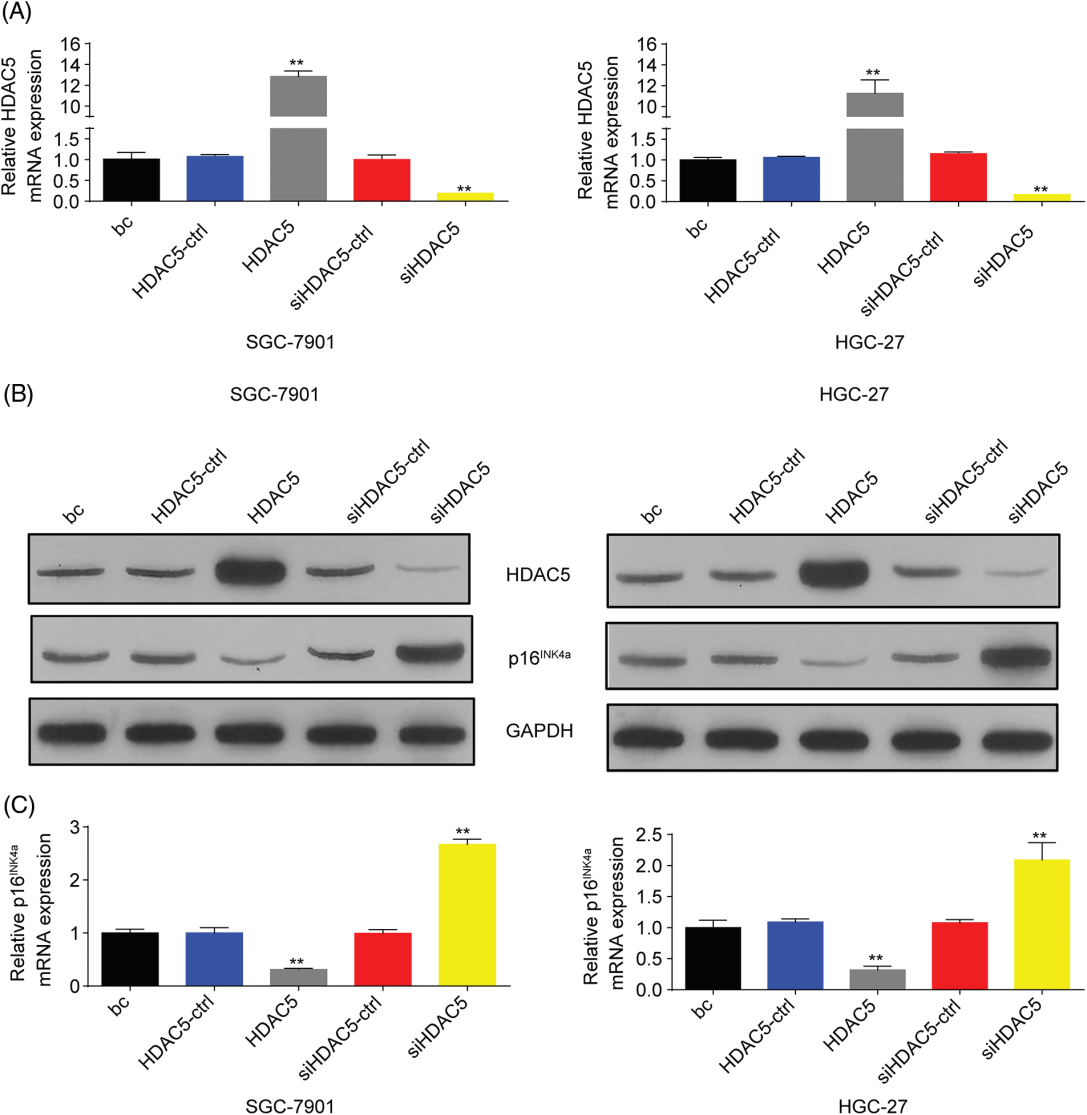
HDAC5 inhibited the expression of p16^INK4a^ in GC cells. (A) The expression of HDAC5 at mRNA levels was detected by RT‒qPCR in SGC-7901 and HGC-27 cells with HDAC5 overexpression or silencing. **p < 0.01 *vs.* HDAC5-ctrl, siHDAC5-ctrl or bc. (B) The inhibition or promoting of p16^INK4a^ and HDAC5 protein expression by HDAC5 overexpression or silencing were confirmed by western blot in SGC-7901 and HGC-27 cells. (C) The expression of p16^INK4a^ at mRNA levels was detected by RT‒qPCR in SGC-7901 and HGC-27 cells with HDAC5 overexpression or silencing. **p < 0.01 *vs.* HDAC5-ctrl, siHDAC5-ctrl or bc. bc, blank control; HDAC5, HDAC5 expression plasmid; HDAC5-ctrl, HDAC5-negative control; siHDAC5, siRNA specific for HDAC5; siHDAC5-ctrl, siHDAC5-negative control. Each experiment was repeated three times.

**Figure 6:**
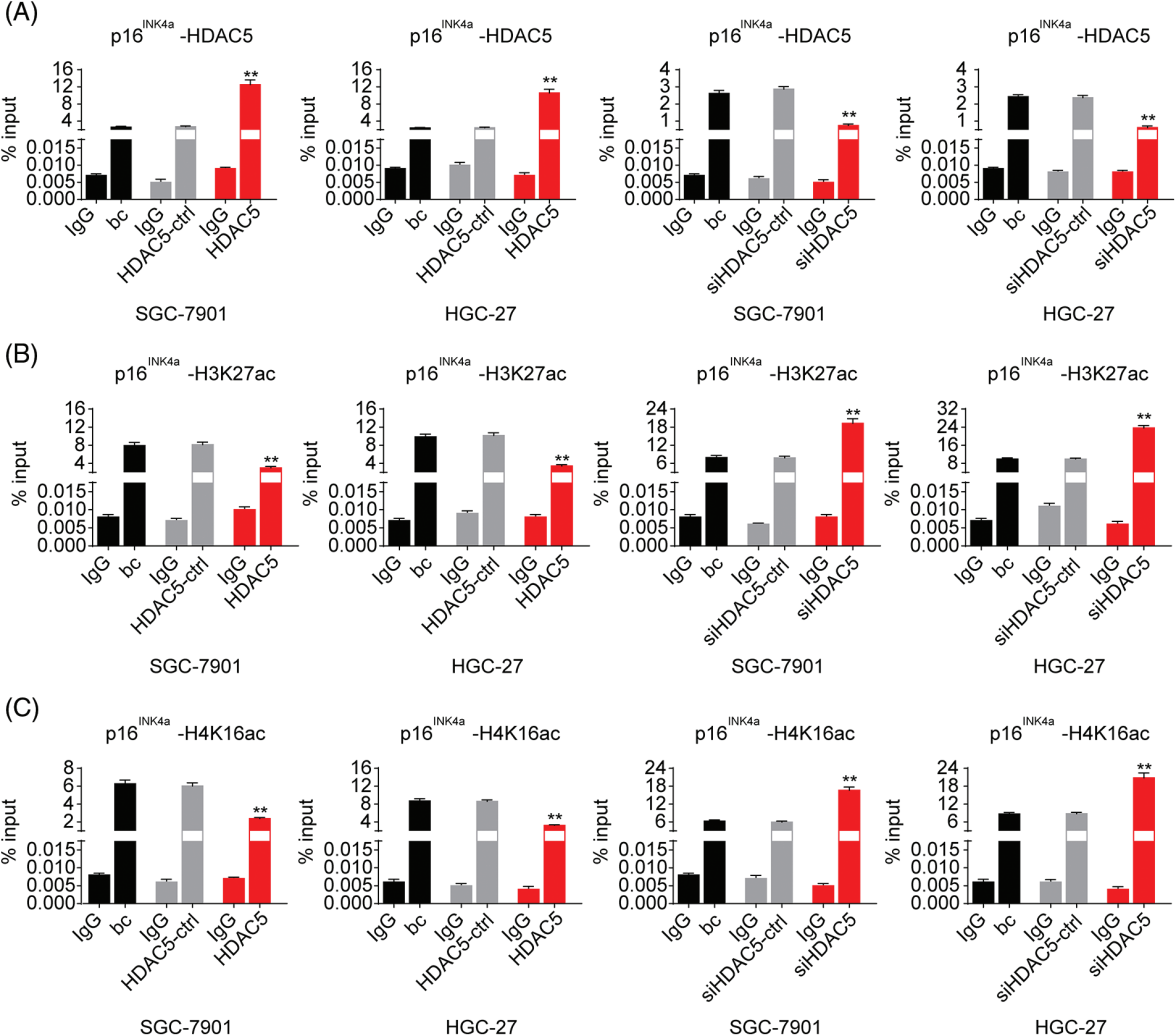
Effects of HDAC5 on the epigenetic modulation of the p16^INK4a^ gene. (A) ChIP results of bindings of HDAC5 to the promoter of the p16^INK4a^ gene following the transiently transfected HDAC5 expression plasmid or siRNA specific for HDAC5 in SGC-7901 and HGC-27 cells. **p < 0.01 *vs.* HDAC5-ctrl, siHDAC5-ctrl or bc. (B) ChIP results of bindings of H3K27ac to the promoter of the p16^INK4a^ gene following the transiently transfected HDAC5 expression plasmid or siRNA specific for HDAC5 in SGC-7901 and HGC-27 cells. **p < 0.01 *vs.* HDAC5-ctrl, siHDAC5-ctrl or bc. (C) ChIP results of bindings of H4K16ac to the promoter of the p16^INK4a^ gene following the transiently transfected HDAC5 expression plasmid or siRNA specific for HDAC5 in SGC-7901 and HGC-27 cells. **p < 0.01 *vs.* HDAC5-ctrl, siHDAC5-ctrl or bc. bc, blank control; HDAC5, HDAC5 expression plasmid; HDAC5-ctrl, HDAC5-negative control; siHDAC5, siRNA specific for HDAC5; siHDAC5-ctrl, siHDAC5-negative control. Each experiment was repeated three times.

To further evaluate the association of HDAC5 with p16^INK4a^ expression, we transiently transfected an HDAC5 expression plasmid (pCMV3-HDAC5) into SGC-7901 and HGC-27 cells. The results showed that the transient overexpression of HDAC5 led to the increased expression of HDAC5 mRNA (Fig. 5A) and protein (Fig. 5B), and the decreased expression of p16^INK4a^ mRNA (Fig. 5C) and protein (Fig. 5B). In addition, siRNA was used to knock down the endogenous expression of HDAC5, and the expression of p16^INK4a^ mRNA and protein was examined in SGC-7901 and HGC-27 cells. The siRNA specific for HDAC5 significantly reduced the expression of HDAC5 mRNA (Fig. 5A) and protein (Fig. 5B), and silencing of HDAC5 caused a significant increase in the expression of p16^INK4a^ mRNA (Fig. 5C) and protein (Fig. 5B). Further results showed that transient transfection of the HDAC5 expression plasmid in SGC-7901 and HGC-27 cells resulted in increased enrichment of HDAC5 (Fig. 6A) and decreased enrichment of the activating histone marks H3K27ac (Fig. 6B) and H4K16ac (Fig. 6C) at the p16^INK4a^ promoter compared with the blank control or the HDAC5-control. Opposite results of HDAC5 (Fig. 6A), H3K27ac (Fig. 6B) and H4K16ac (Fig. 6C) enrichment were observed at the p16^INK4a^ promoter when HDAC5 expression was transiently knocked down in SGC-7901 and HGC-27 cells.

**Figure 7:**
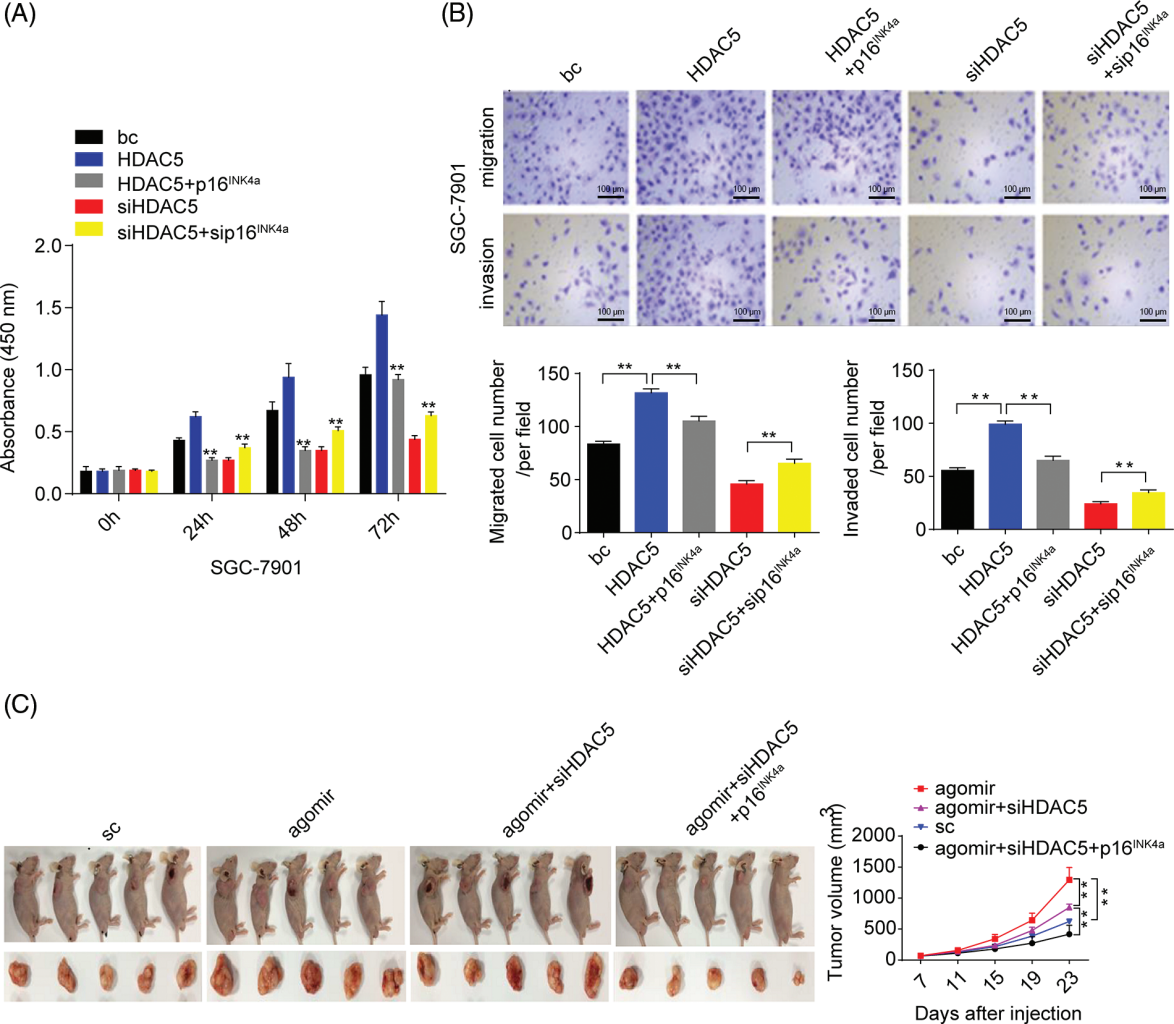
Effects of the miR-4256/HDAC5/p16^INK4a^ axis on GC growth and invasion. (A) Effects of the HDAC5/p16^INK4a^ axis on the proliferation of SGC-7901 cells were determined by CCK-8 assays. **p < 0.01 *vs.* bc, HDAC5 or siHDAC5. (B) Effects of the HDAC5/p16^INK4a^ axis on the migration and invasion of SGC-7901 cells were determined by Transwell assays. **p < 0.01 *vs.* bc, HDAC5 or siHDAC5. (C) Tumour size was monitored and measured with calipers starting from the 7th day after tumour cell inoculation in each group. The pictures of xenograft tumors and grow curves of tumors from miR-4256 agomir, miR-4256 agomir plus siHDAC5, miR-4256 agomir plus siHDAC5 and p16^INK4a^ and sc group. **p < 0.01 *vs.* sc, agomir or agomir plus siHDAC5 and p16^INK4a^. bc, blank control; HDAC5, HDAC5 expression plasmid; p16^INK4a^, p16^INK4a^ expression plasmid; siHDAC5, siRNA specific for HDAC5; sip16^INK4a^, siRNA specific for p16^INK4a^. *p < 0.05, **p < 0.01. Each experiment was repeated three times.

**Figure S6:**
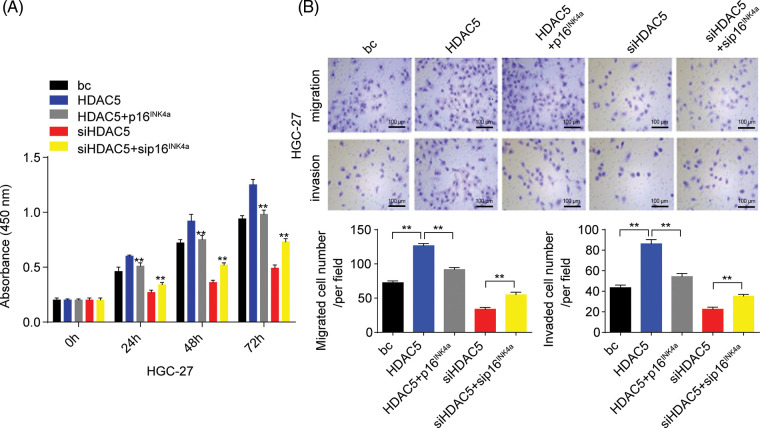
Effects of the HDAC5/p16^INK4a^ axis on malignant biological behavior of GC cells. (A) Effects of the HDAC5/p16^INK4a^ axis on the proliferation of HGC-27 cells were determined by CCK-8 assays. **p < 0.01 *vs.* bc, HDAC5 or siHDAC5. (B) Effects of the HDAC5/p16^INK4a^ axis on the migration and invasion of HGC-27 cells were determined by Transwell assays **p < 0.01 *vs.* bc, HDAC5 or siHDAC5. bc, blank control; HDAC5, HDAC5 expression plasmid; p16^INK4a^, p16^INK4a^ expression plasmid; siHDAC5, siRNA specific for HDAC5; sip16^INK4a^, siRNA specific for p16^INK4a^. Each experiment was repeated three times.

Furthermore, we analyzed the role of HDAC5/p16^INK4a^ signaling in GC cell proliferation, migration, and invasion capacities. The transient overexpression of HDAC5 resulted in increased proliferation and augmented migration and invasion in GC cells (SGC-7901 and HGC-27 cells) compared with the blank control; the combination of the transient overexpression of HDAC5 and p16^INK4a^ abolished the promoting effect of HDAC5 on GC cell proliferation, migration and invasion, while the combination of the transient knockdown of HDAC5 and p16^INK4a^ markedly reversed the inhibitory effect of silencing HDAC5 on GC cell proliferation (Fig. 7A and Suppl. Fig. S6A), migration and invasion (Fig. 7B and Suppl. Fig. S6B) in GC cells (SGC-7901 and HGC-27 cells) compared with the blank control.

We finally investigated the involvement of the miR-4256/HDAC5/p16^INK4a^ signaling axis in the *in vivo* tumorigenicity of GC cells. As shown in Fig. 7C, the tumors in the mice with miR-4256 agomir treatment grew more rapidly compared with the scrambled control; combined intratumoral injection of miR-4256 agomir and cholesterol-modified siHDAC5 partly eliminated the promoting effect of miR-4256 overexpression on tumor growth, while cointratumoral injection of miR-4256 agomir and cholesterol-modified siHDAC5 plus p16^INK4a^ overexpression exhibited stronger tumor growth inhibition (Fig. 7C).

Collectively, these observations suggest that miR-4256 suppresses p16^INK4a^ expression to augment the malignant biological behavior of GC cells through the epigenetic modulation of HDAC5 at the p16^INK4a^ promoter.

### miR-4256 expression is upregulated by the SMAD2/p300 complex in GC cells

The general principle of gene expression regulation is that transcription factors modulate transcription by recruiting transcriptional coactivators or corepressors that modify histones and chromatin structure. The transcriptional coactivator p300 has a large number of interacting protein partners, thereby acting as a hub in gene regulatory networks [24,25]. In addition, p300 also possesses intrinsic histone acetyltransferase activity that acetylates all four core histones to activate gene transcription in nucleosomes [25,26]. Based on the key role of p300 in gene expression regulation, we speculate that p300 may be involved in the overexpression of miR-4256 in GC.

To address the mechanism underlying miR-4256 overexpression in GC, bioinformatics analyses were first performed. The database String (https://cn.string-db.org/) was used to analyze the p300 interactions with transcription factors, and the results showed that 6 transcription factors, including MYOD1, SMAD2, YY1, TP53, PPARG and STAT3 had high combined scores. Furthermore, the database USCS (http://genome.ucsc.edu/) was used to obtain the promoter sequence of miR-4256 (1000 bp range of genomic DNA upstream of the miR-4256 gene), and the database JASPAR (https://jaspar.genereg.net/) was used to analyze the potential transcription factors that can bind to the promoter of miR-4256, indicating that there are two putative binding sites for SMAD2 (site 1 (−738/−726) and site 2 (−792/−776)) at the miR-4256 promoter that have higher scores; therefore, SMAD2 was selected as the candidate transcription factor.

**Figure 8:**
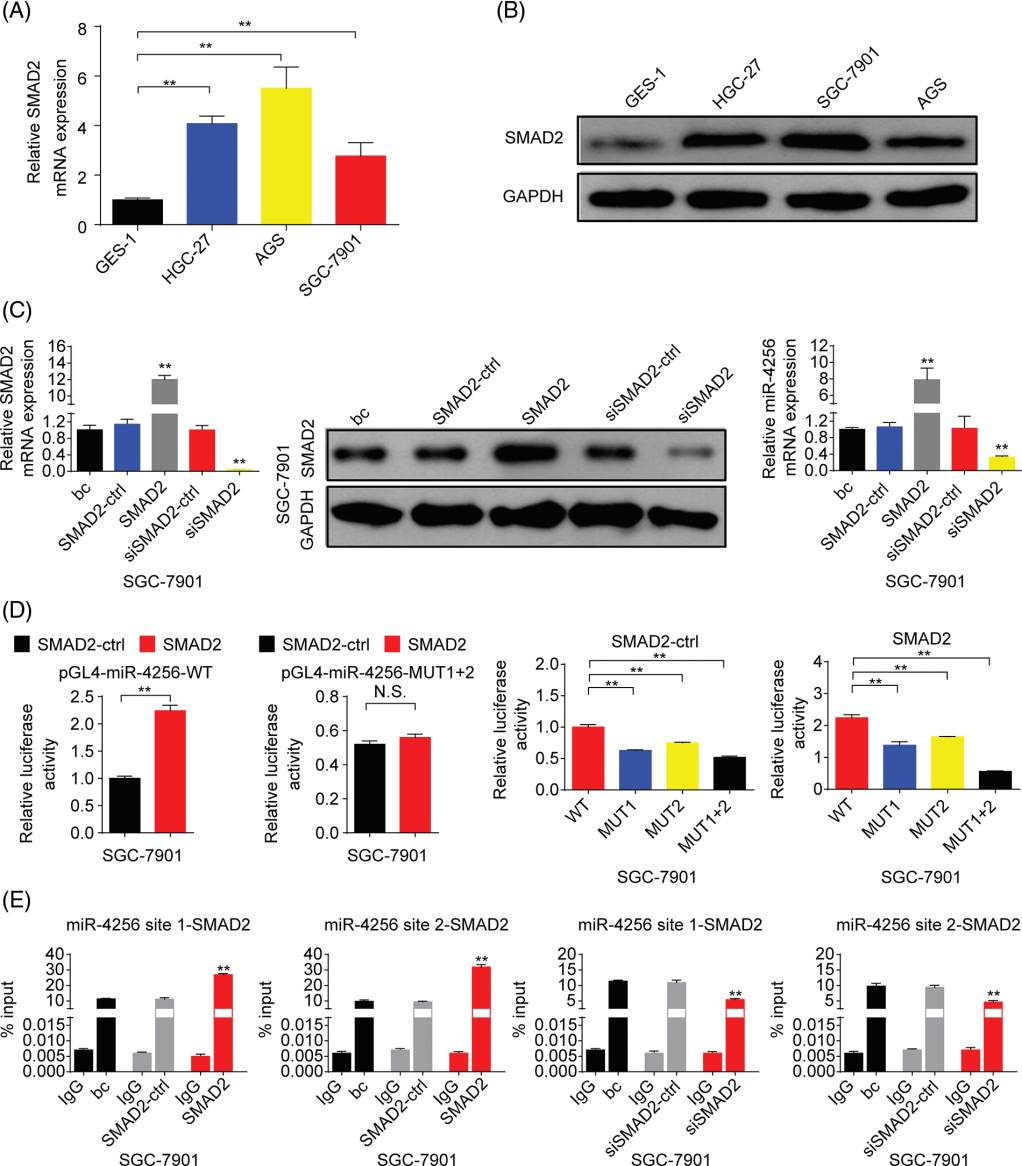
SMAD2 bound to the promoter of the miR-4256 gene to positively regulate miR-4256 expression in GC cells. (A, B) The expression levels of SMAD2 mRNA and protein were detected in GC cells (SGC-7901, HGC-27, and AGS) and human gastric epithelial cell line (GES-1) by RT‒qPCR and western blot. **p < 0.01 *vs.* GES-1 cells. (C) The expression of SMAD2 mRNA and protein and the expression of miR-4256 were detected in SGC-7901cells transiently transfected with the SMAD2 expression plasmid or siRNA specific for SMAD2. **p < 0.01 *vs.* bc, SMAD2 or siSMAD2. (D) Dual-luciferase reporter assays for confirming the direct binding relationship between miR-4256 and SMAD2 in SGC-7901 cells. **p < 0.01. (E) ChIP results of bindings of SMAD2 to the site1 and site2 of the miR-4256 promoter following the transiently transfecting SMAD2 expression plasmid or siRNA specific for SMAD2 in SGC-7901 cells. **p < 0.01 *vs.* bc, SMAD2-ctrl or siSMAD2-ctrl. bc, blank control; SMAD2, SMAD2 expression plasmid; SMAD2-ctrl, SMAD2-negative control; siSMAD2, siRNA specific for SMAD2; siSMAD2-ctrl, siSMAD2-negative control; WT, pGL4-miR-4256-WT; MUT, pGL4-miR-4256-MUT; N.S., no significance. Each experiment was repeated three times.

**Figure S7:**
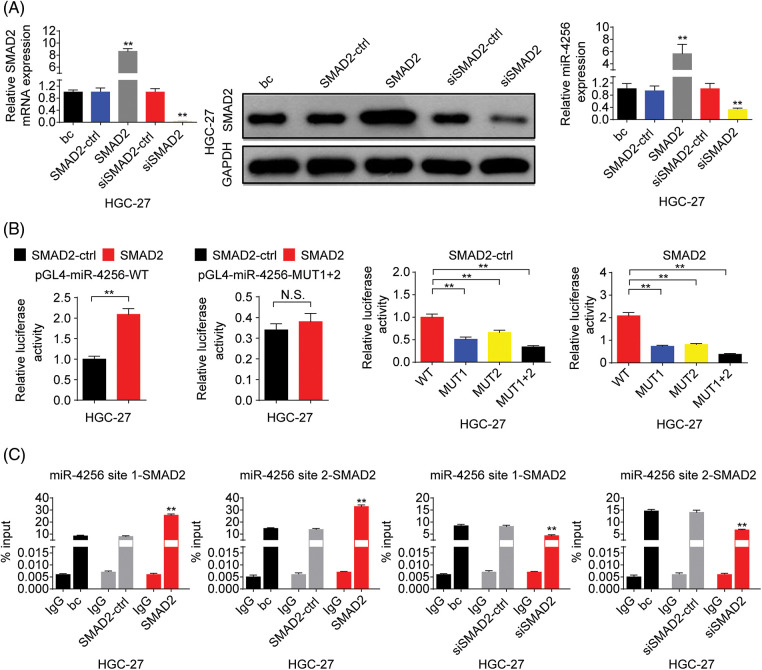
SMAD2 bound to the promoter of the miR-4256 gene to positively regulate miR-4256 expression in GC cells. (A) The expression of SMAD2 mRNA and protein and the expression of miR-4256 were detected in HGC-27 cells transiently transfected with the SMAD2 expression plasmid or siRNA specific for SMAD2. **p < 0.01 *vs.* bc, SMAD2-ctrl or siSMAD2-ctrl. (B) Dual-luciferase reporter assays for confirming the direct binding relationship between miR-4256 and SMAD2 in HGC-27 cells. **p < 0.01. (C) ChIP results of bindings of SMAD2 to the site1 and site2 of the miR-4256 promoter following the transiently transfecting SMAD2 expression plasmid or siRNA specific for SMAD2 in HGC-27 cells. **p < 0.01 *vs.* bc, SMAD2-ctrl or siSMAD2-ctrl. bc, blank control; SMAD2, SMAD2 expression plasmid; SMAD2-ctrl, SMAD2-negative control; siSMAD2, siRNA specific for SMAD2; siSMAD2-ctrl, siSMAD2-negative control; WT, pGL4-miR-4256-WT; MUT, pGL4-miR-4256-MUT; N.S., no significance. Each experiment was repeated three times.

Next, we explored the expression of SMAD2 in GC. The results showed that the expression of SMAD2 mRNA (Fig. 8A) and protein (Fig. 8B) was significantly higher in GC cell lines (SGC-7901, HGC-27, and AGS cells) than in the human gastric epithelial cell line (GES-1). Then, we investigated whether SMAD2 affects miR-4256 expression. The SMAD2 expression plasmid (pCMV3-SMAD2) was transiently transfected into SGC-7901 and HGC-27 cells. The results showed that the transient overexpression of SMAD2 resulted in the increased expression of SMAD2 mRNA and protein, and the increased expression of miR-4256 compared with the blank control or the SMAD2-control (Fig. 8C and Suppl. Fig. S7A). In addition, siRNA was used to knock down the endogenous expression of SMAD2, and miR-4256 expression was examined in SGC-7901 and HGC-27 cells. The siRNA specific for SMAD2 significantly reduced the expression of SMAD2 mRNA (Fig. 5A) and protein (Fig. 5B), and lowered miR-4256 expression compared with the blank control or the siSMAD2-control (Fig. 8C and Suppl. Fig. S7A). We further investigated whether SMAD2 binds to the promoter of miR-4256 to regulate miR-4256 expression. To address this issue, luciferase reporter assays were performed. The results indicated that compared with the SMAD2 negative control, transient overexpression of SMAD2 significantly increased the relative luciferase activity of the the pGL4-miR-4256-WT construct, but the relative luciferase activity of the pGL4-miR-4256-MUT1+2 construct was not significantly affected by transient overexpression of SMAD2 in SGC-7901 (Fig. 8D) and HGC-27 (Suppl. Fig. S7B) cells. In addition, the relative luciferase activity of the pGL4-miR-4256-WT construct was significantly higher than that of pGL4-miR-4256-MUT1+2 construct, pGL4-miR-4256-MUT1 construct, or pGL4-miR-4256-MUT2 construct in SGC-7901 (Fig. 8D) and HGC-27 (Suppl. Fig. S7B) cells transfected with SMAD2 negative control or SMAD2 expression plasmid. To further confirm that SMAD2 could physically bind to the miR-4256 promoter *in vivo*, ChIP assays were performed. The results showed that the enrichment of SMAD2 at the two putative SMAD2 binding sites of the miR-4256 promoter was increased by the transient overexpression of SMAD2 and decreased by the transient knockdown of SMAD2 by siSMAD2 in SGC-7901 (Fig. 8E) and HGC-27 cells (Suppl. Fig. S7C). These observations indicate that the transcription factor SMAD2 can interact directly with the miR-4256 promoter and upregulate miR-4256 expression levels in GC cells.

**Figure 9:**
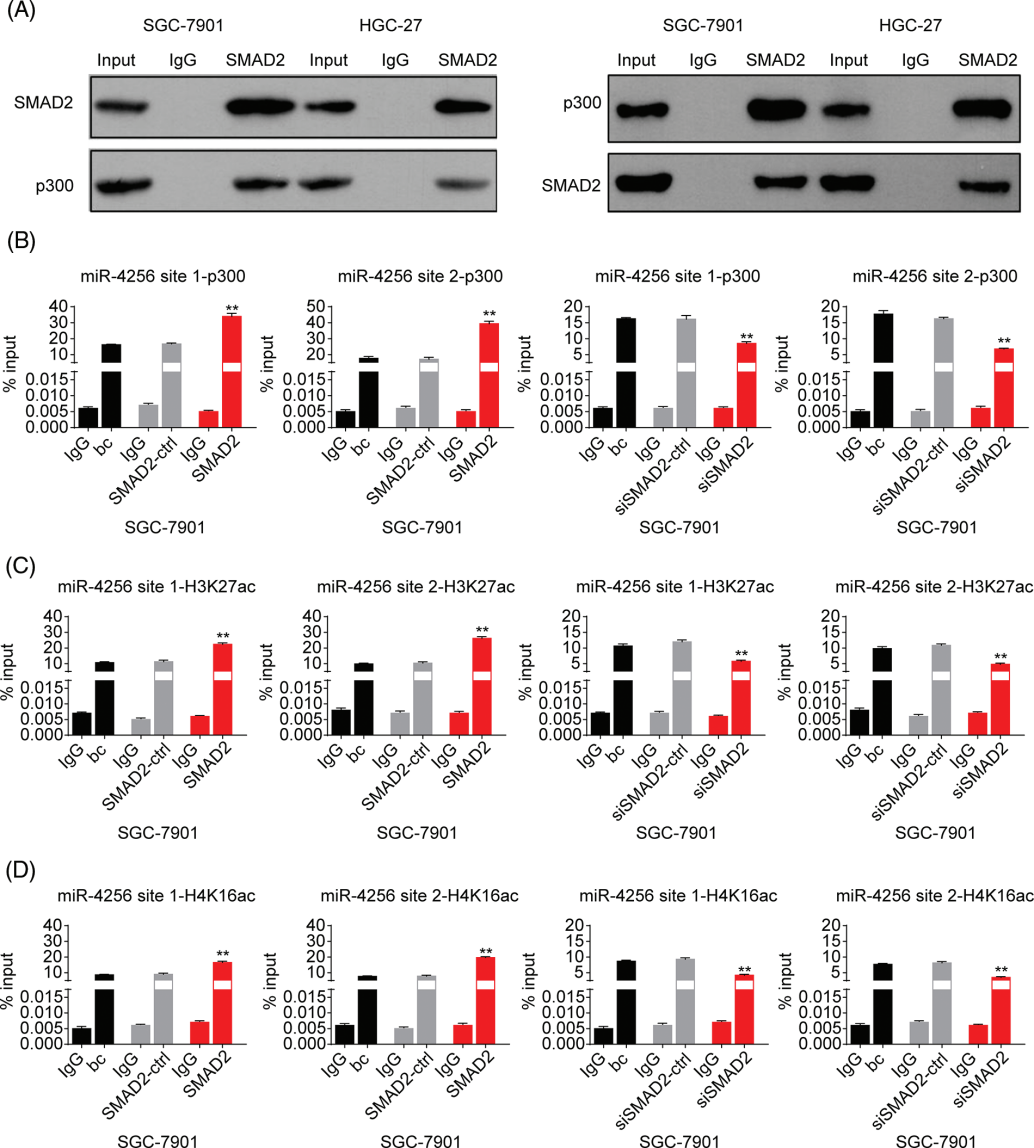
Effects of SMAD2 on the enrichment of p300, H3K27ac, and H4K16ac at the promoter of miR-4256 in GC cells. (A) Coimmunoprecipitation assays showed the presence of a complex containing SMAD2 and p300. (B) ChIP assays demonstrated the enrichment change of p300 to the site1 and site2 of the miR-4256 promoter in SGC-7901 cells. **p < 0.01 *vs.* bc, SMAD2-ctrl or siSMAD2-ctrl. (C) ChIP assays demonstrated the enrichment change of H3K27ac to the site1 and site2 of the miR-4256 promoter in SGC-7901 cells. **p < 0.01 *vs.* bc, SMAD2-ctrl or siSMAD2-ctrl. (D) ChIP assays demonstrated the enrichment change of H4K16ac to the site1 and site2 of the miR-4256 promoter in SGC-7901 cells. **p < 0.01 *vs.* bc, SMAD2-ctrl or siSMAD2-ctrl. bc, blank control; SMAD2, SMAD2 expression plasmid; SMAD2-ctrl, SMAD2-negative control; siSMAD2, siRNA specific for SMAD2; siSMAD2-ctrl, siSMAD2-negative control. Each experiment was repeated three times.

Furthermore, to confirm the bioinformatics analysis results that SMAD2 may interact with p300, we performed coimmunoprecipitation experiments. Cell lysates of GC cells (SGC-7901 and HGC-27 cells) were immunoprecipitated with an anti-SMAD2 antibody, followed by Western blotting with anti-p300 antibody. Reverse co-immunoprecipitation was performed with an anti-p300 antibody, followed by Western blotting with an anti-SMAD2 antibody. The results showed that SMAD2 directly interacted with p300, suggesting SMAD2-p300 complex formation *in vivo* (Fig. 9A).

**Figure S8:**
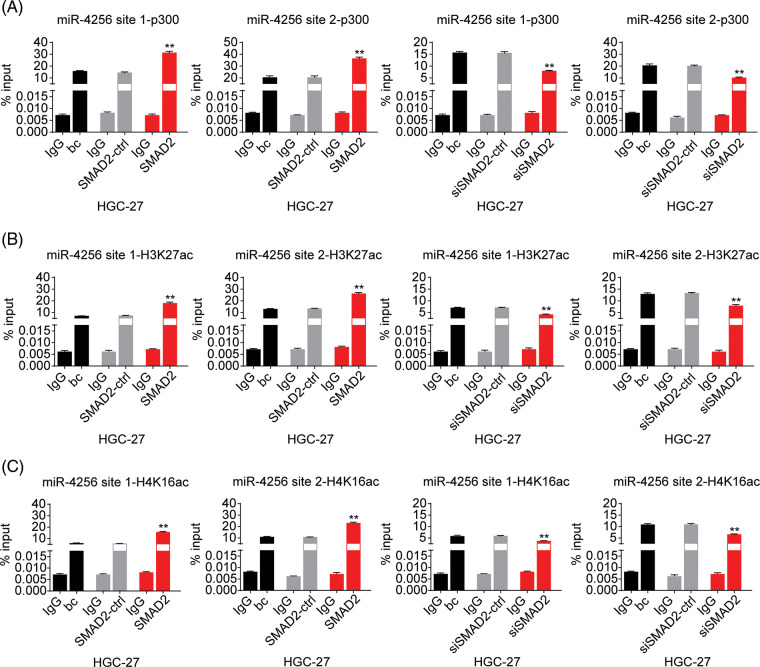
Effects of SMAD2 on the enrichment of p300, H3K27ac, and H4K16ac at the promoter of the miR-4256 in GC cells. (A) ChIP assays showed the enrichment change of p300 to the site1 and site2 of the miR-4256 promoter in HGC-27 cells. **p < 0.01 *vs.* bc, SMAD2-ctrl or siSMAD2-ctrl. (B) ChIP assays showed the enrichment change of H3K27ac to the site1 and site2 of the miR-4256 promoter in HGC-27 cells. **p < 0.01 *vs.* bc, SMAD2-ctrl or siSMAD2-ctrl. (C) ChIP assays showed the enrichment change of H4K16ac to the site1 and site2 of the miR-4256 promoter in HGC-27 cells. **p < 0.01 *vs.* bc, SMAD2-ctrl or siSMAD2-ctrl. bc, blank control; SMAD2, SMAD2 expression plasmid; SMAD2-ctrl, SMAD2-negative control; siSMAD2, siRNA specific for SMAD2; siSMAD2-ctrl, siSMAD2-negative control. Each experiment was repeated three times.

Finally, ChIP assays were performed to explore the effects of SMAD2 on the enrichment of p300 and activating histone marks H3K27ac and H4K16ac at the promoter of miR-4256 in GC cells. The results showed that the enrichment of p300 (Fig. 9B and Suppl. Fig. S8A), H3K27ac (Fig. 9C and Suppl. Fig. S8B), and H4K16ac (Fig. 9D and Suppl. Fig. S8C) at the two putative SMAD2 binding sites of the miR-4256 promoter in SGC-7901 and HGC-27 cells were increased by the transient overexpression of SMAD2 and decreased by the transient knockdown of SMAD2 by siSMAD2.

Taken together, these results suggest that SMAD2 binds to the miR-4256 promoter and increases the recruitment of p300 to this promoter by interacting with p300, further resulting in the enrichment of activating histone marks H3K27ac and H4K16ac and thus upregulating miR-4256 expression.

### SMAD2/miR-4256 signaling modulates GC cell proliferation, migration, and invasion capacities

**Figure 10:**
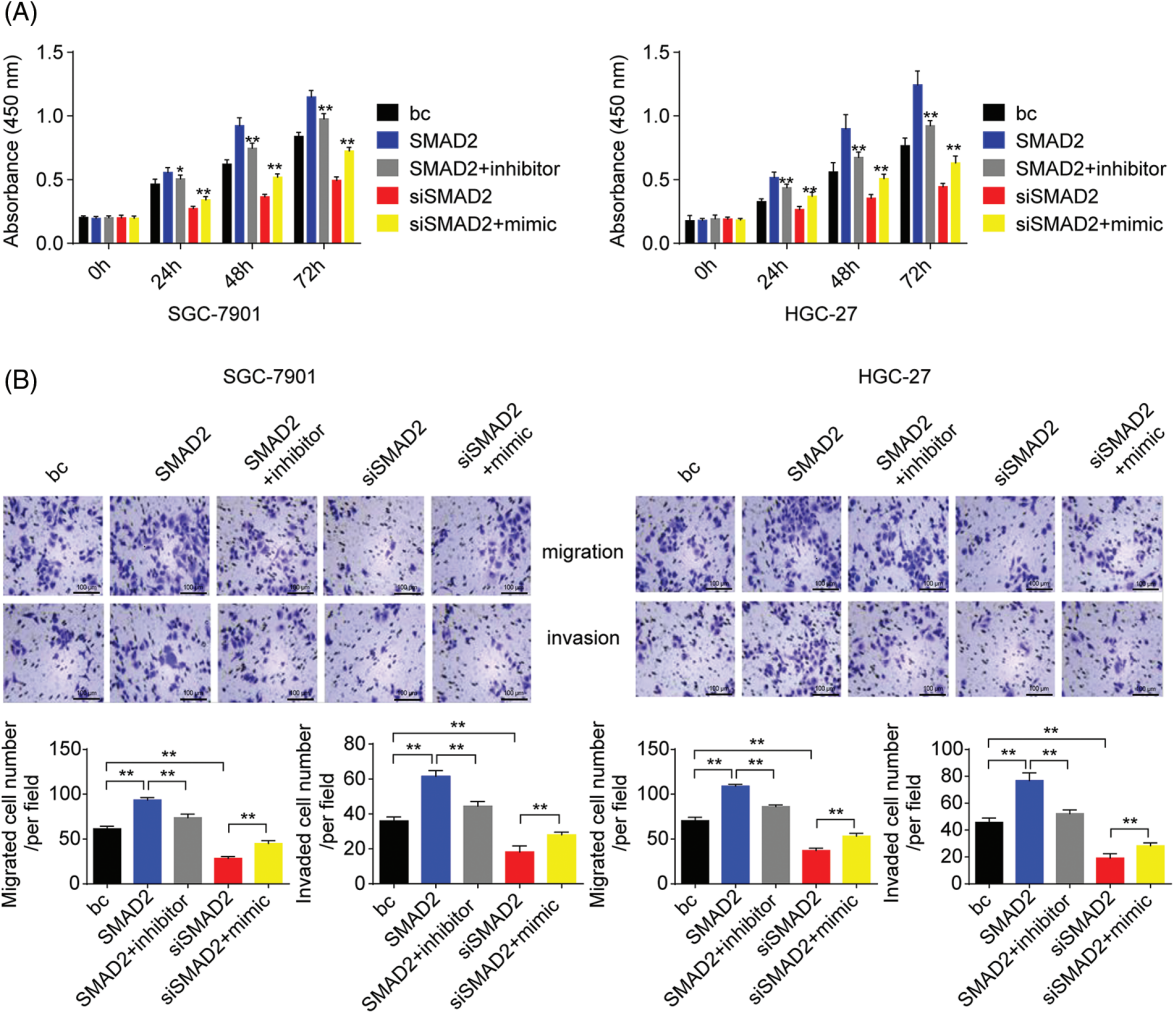
Effects of the SMAD2/miR-4256 axis on GC cell growth and invasion. (A) Effects of the SMAD2/miR-4256 axis on the proliferation of SGC-7901 and HGC-27 cells were determined by CCK-8 assays. **p < 0.01 *vs.* bc, SMAD2 or siSMAD2. (B) Effects of the SMAD2/miR-4256 axis on the migration and invasion of SGC-7901 and HGC-27 cells were determined by Transwell assays. **p < 0.01 *vs.* bc, SMAD2 or siSMAD2. bc, blank control; SMAD2, SMAD2 expression plasmid; siSMAD2, siRNA specific for SMAD2; mimic, miR-4256 mimic; inhibitor, miR-4256 inhibitor. Each experiment was repeated three times.

To further assess the role of SMAD2/miR-4256 signaling in GC cell proliferation, migration, and invasion, CCK-8 and Transwell assays were performed. The results showed that the transient overexpression of SMAD2 enhanced the proliferation, migration, and invasion of GC cells (SGC-7901 and HGC-27 cells) compared with the blank control; of note, the combination of the transient transfection of SMAD2 and miR-4256 inhibitor could partly eliminate the promoting effect on the malignant biological behavior caused by SMAD2 overexpression. In contrast, transient SMAD2 knockdown by siRNA attenuated proliferation, migration, and invasion in GC cells (SGC-7901 and HGC-27 cells) compared with the blank control, while the combination of transient SMAD2 knockdown and transient miR-4256 overexpression markedly reversed the inhibitory effect of silencing SMAD2 on GC cell proliferation (Fig. 10A), migration and invasion (Fig. 10B).

Collectively, these results indicate that the SMAD2/p300 complex upregulates miR-4256 expression to augment the malignant biological behavior of GC cells.

## Discussion

In the current study, we provide for the first time comprehensive and in-depth knowledge of the critical role of a newly identified serum exosomal miRNA miR-4256, which is a 64 nt small noncoding RNA molecule that is approximately three times longer than common miRNAs, in the progression of GC via the SMAD2/miR-4256/HDAC5/p16^INK4a^ axis. We report that miR-4256 was overexpressed in GC tissues and cell lines. Our results further revealed that miR-4256 promoted proliferation, migration and invasion *in vitro* and stimulated tumorigenesis *in vivo* by enhancing the repressive effect of HDAC5 on p16^INK4a^ expression by decreasing the enrichment of H3K27ac and H4K16ac of the p16^INK4a^ promoter. Moreover, miR-4256 expression was upregulated by the SMAD2/p300 complex in GC cells. These data indicate that the SMAD2/miR-4256/HDAC5/p16^INK4a^ axis is a novel oncogenic pathway and that targeting it might be a potential treatment strategy for GC.

Recently, increasing evidence has suggested that exosomal miRNAs are frequently dysregulasted in a wide range of cancers and widely involved in cancer initiation and progression, especially GC [27,28]. In this study, we performed next-generation sequencing to identify differentially expressed miRNAs within serum exosomes of GC patients. We selected miR-4256, an upregulated miRNA with the highest fold-change and a significant *p* value in miRNA profiles, to explore its regulatory role in GC. Our results showed that miR-4256 was highly expressed in GC cell lines but weakly expressed in a human gastric epithelial cell line. The expression of miR-4256 was also higher in GC tissues than in adjacent nontumor tissues. Moreover, clinicopathological characteristics indicated that high expression of miR-4256 has a positive association with lymph node metastasis, later TNM stage (stages III–IV), and poorly differentiated tumor tissues of GC patients. Based on these results, we concluded that miR-4256 may function as an oncogene and play vital roles in the development and progression of GC. Furthermore, functional experiments demonstrated that miR-4256 overexpression promoted the proliferation, migration and invasion of GC cells and that miR-4256 knockdown reduced the proliferation, migration and invasion of GC cells; *in vivo* experiments demonstrated that miR-4256 overexpression resulted in the faster growth of tumors in a nude mouse model. These results confirm the tumor-promoting activity of miR-4256 in GC and provide evidence of the potential utility of this miRNA as a prognostic indicator and therapeutic target of GC.

Next, we investigated the potential mechanisms of miR-4256-mediated GC promotion. The HDAC5 gene is a member of the HDAC family of enzymes, and plays vital roles in the tumor progression of multiple human cancers including GC [29]. Liao et al. confirmed that HDAC5 was highly expressed in GC tumor tissues and promoted the proliferation and migration capability of GC cells [30]. Moreover, Lin et al. showed that HDAC5 was upregulated in GC cells and boosted the invasiveness of GC cells by enhancing matrix metalloproteinase 9 (MMP9) [31]. MMP9 has been confirmed as an important gene implicated in tumor invasion and metastasis through epithelial-mesenchymal transition (EMT) processes [32]. In this regard, enforced miR-589-5p expression promoted proliferation, migration and invasion *in vitro*, whereas HDAC5 depletion counteracted the promoting effect on malignant phenotypes [33]. Consistently, we identified that HDAC5 was a direct target of miR-4256 in GC. First, the HDAC5 mRNA level was significantly upregulated in human GC tissues and positively associated with miR-4256 expression. Second, miR-4256 positively regulated the expression of HDAC5 mRNA and protein. Third, miR-4256 expression was detected in both the nucleus and cytoplasm. Fourth, the ectopic overexpression of miR-4256 obviously increased the activity of a luciferase reporter containing the wild-type HDAC5 promoter. Fifth, the transient overexpression of HDAC5 in miR-4256-downregulated GC cells eliminated the inhibition of the malignant biological behavior caused by the miR-4256 inhibitor. These results indicated that miR-4256 could transcriptionally stimulate HDAC5 expression by binding to the promoter of the HDAC5 gene, thus promoting GC cell proliferation, migration and invasion. Previous studies have also reported that several miRNAs such as miR-2861 and miR-9 posttranscriptionally regulate HDAC5 by targeting the coding sequence and 3′UTR of HDAC5 mRNA, respectively [34,35]. Additionally, Ogryzko et al. showed that miR-558 induced the transcriptional activation of heparanase gene through the binding site within its promoter; Ortega et al. showed that miR-337-3p directly bound the matrix metalloproteinase 14 (MMP-14) promoter to suppress its transcription [36,37]. These results support our findings that miR-4256 promotes HDAC5 expression to exacerbate malignant progression in GC by targeting the promoter of the HDAC5 gene. To our knowledge, our study is the first to demonstrate the function of miR-4256 as a vital transcriptional regulator of HDAC5 in GC cells.

p16^INK4a^, a cell cycle regulator encoded by the CDKN2A gene, controls the G1 phase of the cell cycle to S phase through the cyclin-dependent kinase-4 and -6/retinoblastoma (CDK4/6/Rb) pathway [38]. p16^INK4a^, which acts as a tumor suppressor, is inactivated in diverse human cancers and then promotes carcinogenesis in humans [39]. Although the functions of the p16^INK4a^ gene in tumorigenesis have been studied extensively, limited information is known about its regulation. Li et al. previously showed that miR-877-3p activated the expression of p16 by directly binding to the p16^INK4a^ promoter [40]. Moreover, Reda et al. revealed that miR-22 enhanced the expression levels of p16^INK4a^ by attenuating the methylation levels of the p16^INK4a^ promoter regions [41]. In our current study, we demonstrated the downregulated expression of p16^INK4a^ in human GC tissues and a negative correlation with the expression of miR-4256, and validated the regulatory relationship between miR-4256 and p16^INK4a^ in GC cells. Subsequently, we explored the mechanism underlying miR-4256-mediated p16^INK4a^ downregulation. Here, we showed that miR-4256 could not directly regulate p16^INK4a^ expression in a bioinformatics analysis. As we clearly demonstrated that miR-4256 significantly induced HDAC5 expression in GC cells and Feng et al. reported that HDAC3 restrained p16^INK4a^ expression by binding to the promoter of p16^INK4a^ [42], we hypothesized that miR-4256 might regulate p16^INK4a^ expression via the epigenetic regulation mechanism involving HDAC5. As expected, ChIP assays proved that knockdown of miR-4256 expression resulted in decreased enrichment of HDAC5 and increased enrichment of activating histone marks H3K27ac and H4K16ac at the p16^INK4a^ promoter in GC cells. In the following steps, we also revealed that transient overexpression of HDAC5 led to the decreased expression of p16^INK4a^, accompanied by the increased enrichment of HDAC5 and the decreased enrichment of the activating histone marks H3K27ac and H4K16ac in the p16^INK4a^ promoter. Opposite results were observed when HDAC5 siRNA was transiently transfected. Noticeably, our study is the first report the role of HDAC5 in the regulation of p16^INK4a^ expression in GC cells, thus providing a novel HDAC5-mediated pathway in cancer, in addition to the TAp63/maspin, HIPK2/HIF1α, and p65/NF-κB pathways [29,43,44]. In the final step, the involvement of the miR-4256/HDAC5/p16^INK4a^ signaling axis in the tumorigenicity of GC cells was verified in nude mouse models.

Because accumulating evidence has revealed vital roles of p300 in gene expression regulation, we speculated that p300 might participate in the upregulation of miR-4256 in GC [24–26]. Further bioinformatics analysis indicated that the transcription factor SMAD2 could interact with both p300 and the promoter of miR-4256. SMAD2, which is a pivotal mediator in the activation of the TGF-β signaling pathway, participates in the transcriptional response [45]. In fact, SMAD2 exhibits overexpression in many types of human cancers, including GC, and functions as an oncogene by stimulating cell proliferation and tumor metastasis [46–48]. In our study, we demonstrated that the transcription factor SMAD2 was highly expressed in GC cells. Using datasets from TCGA, we also confirmed the increased expression of SMAD2 in GC tissues and revealed a positive correlation between high SMAD2 expression and a lower overall survival rate in GC patients. Furthermore, SMAD2 positively regulated miR-4256 expression in GC cells. Furthermore, a dual luciferase reporter assay showed that SMAD2 could directly bind to both site 1 (−738/−726) and site 2 (−792/−776) of the miR-4256 promoter region and thus activate its transcription. Subsequently, we performed ChIP experiments and validated that there was a direct interaction between SMAD2 and miR-4256 *in vivo*, which was similar to the previous results by Tong et al. [49]. Thus, SMAD2 is a novel transcriptional activator of miR-4256 expression.

**Figure 11:**
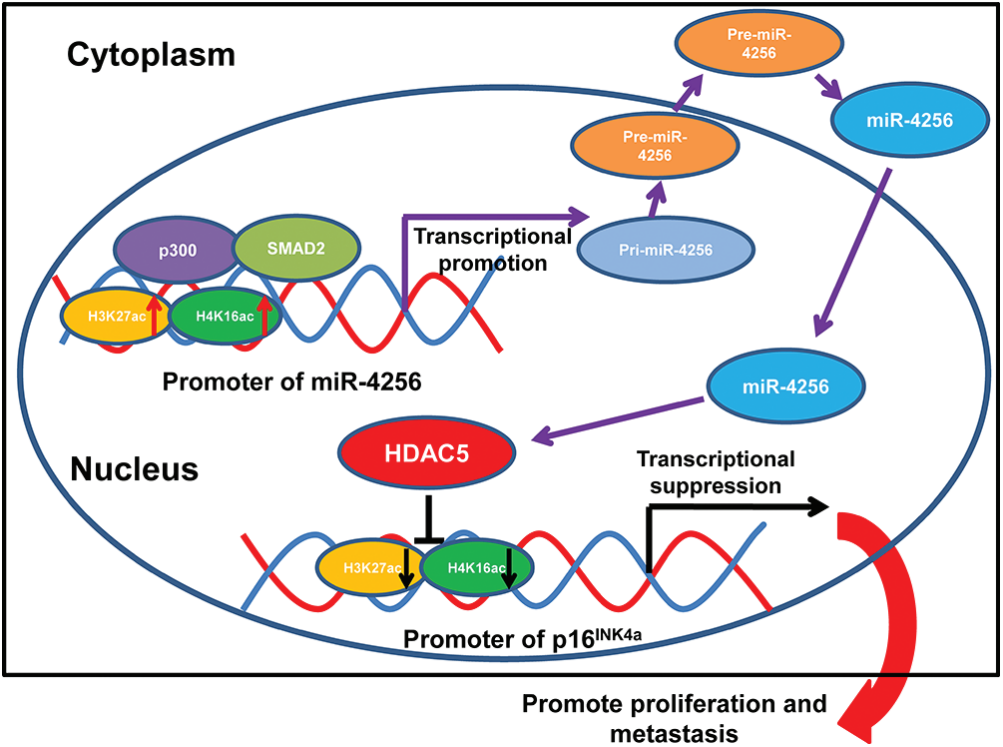
Illustration of the hypothesised signal mechanism of miR-4256.

Formerly, loss-of-function mutations in p300 have been reported to be associated with the development of several types of cancer [50–52]. p300 also functions as a transcriptional coactivator by forming a multiprotein complex with transcription factors and HDACs [53,54]. Moreover, a recent global chromatin profiling study showed that p300 catalyzes lysine acetylation of histone H3 at lysines 27 and 18 (H3K27ac and H3K18ac) in a number of cancer cell lines derived from different cancer types [55]. In our case, we confirmed that SMAD2 and p300 could coprecipitate with each other by coimmunoprecipitation experiments. Moreover, ChIP assays indicated that the ectopic expression of SMAD2 enhanced the enrichment of p300, H3K27ac and H4K16ac at the miR-4256 promoter in GC cells and that the transient knockdown of SMAD2 had the opposite effect on the enrichment of p300, H3K27ac and H4K16ac, suggesting that SMAD2 cooperates with p300 to regulate miR-4256 promoter transcription. This result was supported by previous findings showing that recruitment of p300 increased the enrichment of the activating histone marks H3K27ac, leading to transcriptional activation of the pluripotency factor genes NANOG, SOX2 and KLF4 [56]. Functionally, we showed that SMAD2 overexpression enhanced cell proliferation, migration and invasion and that inhibition of miR-4256 partly abolished the promoting effect of SMAD2 on GC; in addition, SMAD2 knockdown attenuated malignant biological behavior, and miR-4256 overexpression markedly reversed the inhibitory effect of silencing SMAD2 on GC cell proliferation, migration, and invasion. Taken together, the findings presented in this study suggest that the p300/SMAD2/miR-4256/HDAC5/p16^INK4a^ signaling axis plays a vital role in GC development (Fig. 11).

It is well known, that miRNA generally regulates gene expression through hybridization to target the 3′UTR of mRNAs at the posttranscriptional level [57,58]. In addition, miRNA recognition of gene promoters has recently been considered a general mechanism for gene regulation at the transcriptional level [59–63]. Although the underlying molecular mechanism is largely unknown, it may be related to Argonaute (AGO) proteins and/or RNA polymerase II [59–63]. One limitation of this study is that we only preliminarily explored the regulatory role of miR-4256 on HDAC5 in GC cells and mouse models. Further experiments are required to fully elucidate the precise mechanisms of miR-4256-mediated HDAC5 overexpression in GC. Future work is also needed to further substantiate exosomal miR-4256 as a therapeutic and prognostic marker for GC patients.

Notably, the presence of miR-4256 in GC progression reported recently by Zhang et al. was an obvious written error [64]. After reading the full text of this article carefully, we only found miR-4526, not miR-4256, in Fig. 3 (miRNA‒mRNA network) and Fig. 5 (TF-mRNA‒miRNA-lncRNA network). We have contacted the authors, who confirmed that they mistakenly wrote miR-4526 as miR-4256.

In summary, we demonstrate in this article, for the first time, that miR-4256 suppresses p16^INK4a^ expression to augment the malignant biological behavior of GC cells through the epigenetic modulation of HDAC5 at the p16^INK4a^ promoter. Moreover, miR-4256 is positively regulated by the SMAD2/p300 complex in GC cells. Our study not only identifies a novel cancer-promoting miRNA-mediated signaling pathway in GC, but also provides new insights into the pathogenesis of gastric oncogenesis and will aid the development of novel therapeutic and prognostic biomarkers.

## Data Availability

The datasets generated and/or analysed during the current study are available in the ArrayExpress database (accession: E-MTAB-12763, https://www.ebi.ac.uk/arrayexpress/).
